# Milk and dairy consumption and risk of cardiovascular diseases and all-cause mortality: dose–response meta-analysis of prospective cohort studies

**DOI:** 10.1007/s10654-017-0243-1

**Published:** 2017-04-03

**Authors:** Jing Guo, Arne Astrup, Julie A. Lovegrove, Lieke Gijsbers, David I. Givens, Sabita S. Soedamah-Muthu

**Affiliations:** 1 Institute for Food, Nutrition and Health, University of Reading, Reading, RG6 6AR UK; 2 Department of Nutrition, Exercise and Sports, University of Copenhagen, 2200 Copenhagen, Denmark; 3 Hugh Sinclair Unit of Human Nutrition, Institute for Cardiovascular and Metabolic Research, University of Reading, Reading, RG6 6AP UK; 4 Division of Human Nutrition, Wageningen University and Research, 6708 WE Wageningen, The Netherlands

**Keywords:** Dairy, Milk, Fermented dairy, All-cause mortality, Cardiovascular disease, Dose–response meta-analysis

## Abstract

With a growing number of prospective cohort studies, an updated dose–response meta-analysis of milk and dairy products with all-cause mortality, coronary heart disease (CHD) or cardiovascular disease (CVD) have been conducted. PubMed, Embase and Scopus were searched for articles published up to September 2016. Random-effect meta-analyses with summarised dose–response data were performed for total (high-fat/low-fat) dairy, milk, fermented dairy, cheese and yogurt. Non-linear associations were investigated using the spine models and heterogeneity by subgroup analyses. A total of 29 cohort studies were available for meta-analysis, with 938,465 participants and 93,158 mortality, 28,419 CHD and 25,416 CVD cases. No associations were found for total (high-fat/low-fat) dairy, and milk with the health outcomes of mortality, CHD or CVD. Inverse associations were found between total fermented dairy (included sour milk products, cheese or yogurt; per 20 g/day) with mortality (RR 0.98, 95% CI 0.97–0.99; I^2^ = 94.4%) and CVD risk (RR 0.98, 95% CI 0.97–0.99; I^2^ = 87.5%). Further analyses of individual fermented dairy of cheese and yogurt showed cheese to have a 2% lower risk of CVD (RR 0.98, 95% CI 0.95–1.00; I^2^ = 82.6%) per 10 g/day, but not yogurt. All of these marginally inverse associations of totally fermented dairy and cheese were attenuated in sensitivity analyses by removing one large Swedish study. This meta-analysis combining data from 29 prospective cohort studies demonstrated neutral associations between dairy products and cardiovascular and all-cause mortality. For future studies it is important to investigate in more detail how dairy products can be replaced by other foods.

**Electronic supplementary material:**

The online version of this article (doi:10.1007/s10654-017-0243-1) contains supplementary material, which is available to authorized users.

## Introduction

Cardiovascular disease (CVD) is the leading cause of mortality and disability worldwide [1]. Together with smoking, obesity and inactivity, diet is considered to be one of the most important prevention strategies for CVD [2]. Milk and dairy foods have been recommended in most dietary guidelines around the world, but the association of milk or dairy food consumption with CVD is still controversial [3, 4]. An earlier meta-analysis [5] which included 17 prospective cohort studies showed that milk intake was not associated with total mortality or CHD mortality, but there was a borderline significant inverse association with CVD mortality based on limited studies. There were not enough data to examine the effects of other dairy products or milk fat content. Since then, further prospective cohort studies have been published. For example, one recent Swedish publication with two large Swedish cohorts [6] reported that higher milk consumption was associated with a doubling of mortality risk including CVD mortality in the cohort of women. Since this paper was published in 2014, there has been mounting debate from different researchers regarding its seemingly contradictory results [7, 8]. This has caused new uncertainty about the effects of milk and dairy intake on human health. Recently, new meta-analyses of dairy consumption and risk of stroke [9], butter and risk of CVD, diabetes and mortality [10] have been published, showing predominantly neutral or marginally beneficial associations for all dairy products. Therefore, we conducted a comprehensive dose–response meta-analysis to examine linear and non-linear associations between milk and dairy products with all-cause mortality, CHD and CVD events using existing prospective cohort studies of adequate quality.

## Methods

### Literature search and study selection

**Fig. 1:**
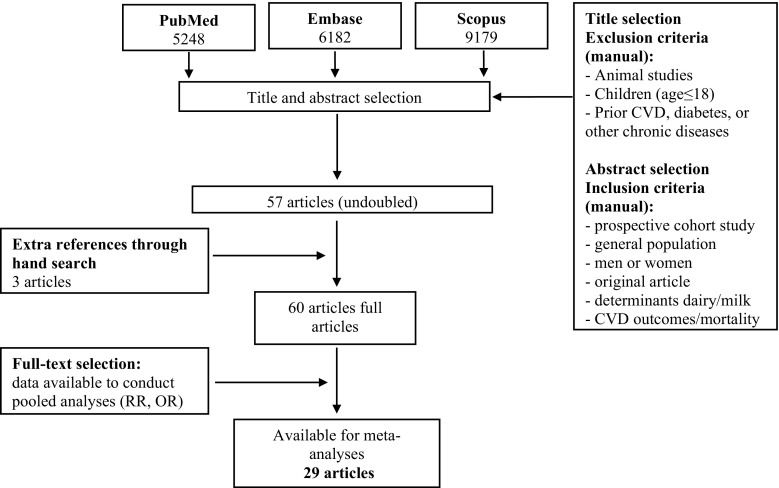
Flowchart of meta-analysis on dairy consumption and incident CVD, CVD mortality and all-cause mortality

This review was conducted based on guidelines of Meta-analysis of Observational Studies in Epidemiology [11]. Prospective cohort studies published up to Sep 2016 (without language restriction) were searched using PubMed, Embase, and Scopus database, the query syntax of searching is shown in the Supplemental Methods (see search strategy). After excluding duplicates and based on titles and abstracts, we excluded studies on animals, baseline age ≤18 years, or populations with prior CVD, diabetes, or any other chronic diseases. Eligible studies were selected by using predefined inclusion criteria of prospective cohort studies, healthy populations and original articles on the association of milk and dairy intake and all-cause mortality, CHD or CVD. In addition, supplementary hand searching of reference lists of previous reviews or meta-analyses was conducted. Of 59 eligible full articles, 29 articles [6, 12–39] met the inclusion criteria (see Fig. 1). Several authors or coworkers provided additional data for this meta-analysis [14, 16, 19, 23, 27, 28, 32, 34, 37, 40].

### Data extraction and quality assessment

Data were extracted from published articles by using a structured extraction form, which included descriptive characteristics of the study, range of intake, median intake, number of participants, number of mortalities, CHD or CVD cases, person-years at risk, and relative risk (RR) with 95% CI for each unit of dairy intake. For studies that reported results from different multivariable-adjusted models, the model with the most confounding factors was extracted for the meta-analysis. If dairy intake was presented in servings or times per period of time [12–20, 22, 23, 34–36, 39], we converted the portion size into grams per day by using standard units of 244 g for milk (585 g for 1 pint of milk); 244 g for yoghurt and 40 g for cheese [41, 42]. One serving of total dairy, high-fat dairy and low-fat dairy was taken to be 200 g, similar to our previous meta-analysis [5]. When studies reported country specific conversion factors, these were used to calculate intake as g/day [26, 29, 30].

In some studies the mean intakes of dairy categories were not reported, in which case we calculated the mean value by using the lower and upper limit. For open-ended upper limits of intake, the same range as the lower category was applied. The categories of dairy types were defined in accordance with the definition in the original articles (Supplemental Table 2).

Two independent reviewers determined the quality of the 29 studies based on the Newcastle–Ottawa quality assessment scale (NOS, Supplemental Methods) [43]. By evaluation of selection, comparability and outcome, the rating system scores studies from 0 (highest degree of bias) to 9 (lowest degree of bias). Additionally we investigated the funding sources of all of the eligible studies. The four categories of funding were recorded as industry, partial funded by industry, research institution and unknown.

### Statistical analysis

Meta-analyses of each dairy type were performed if the number of studies was three or more. Splined variables were generated by MKSPLINE in STATA version 13.0 to determine the most appropriate knot points of nonlinear associations from goodness-of-fit tests and Chi square statistics. Spine analysis and dose–response generalised least-square trend (GLST) meta-analysis were applied for the further analysis of linear or nonlinear associations. Incremental dose–response RRs were derived from the random-effects meta-regression trend estimation of summarised dose–response data. Ding’s spaghetti plot was used to present the shapes of the association within individual studies, as described previously [44]. Forest plots were created to assess the linear dose–response slopes and corresponding 95% CI across relative studies with increments of 200 g/day for total, high-fat, and low-fat dairy; 244 g/day for milk; 20 g/day for total fermented dairy (includes cheese, yogurt and soured milk products); 10 g/day for cheese; 50 g/day for yogurt. Sensitivity analysis was based on linear dose–response slopes by excluding one study population at a time.

To explore heterogeneity between studies, I-squared was calculated from Cochrane Q test [45]. In addition, sub-group analyses were performed providing that at least 6 study populations were available by age (≤50 years, >50 years), follow-up duration (≤10 years, >10 years), gender (men, women, both men and women), continent, confounding factors (whether analyses were or were not adjusted for the following 7 confounders age, sex, smoking, alcohol, body mass index (BMI), physical activity, food energy intake), BMI (≤25 kg/m^2^, >25 kg/m^2^) and Newcastle–Ottawa quality score < or ≥7. When number of the examined studies ≥10, potential publication bias was assessed by means of the Eggers test [46] and symmetry of the funnel plot. All of the statistical analyses were performed in STATA version 13.0 (StataCorp. College Station, Texas, USA). Two-sided *P* values <0.05 were considered as statistically significant.

## Results

Overviews of key characteristics of the 29 prospective cohort studies are shown in Table 1. The included participants of each dairy exposure data on all-cause mortality, CHD or CVD are presented in Table 2. A total of 783,989 participants, 93,158 mortality cases, 28,419 CHD and 25,416 CVD were included in the analysis. There were 3 studies conducted in Asia (Japan and Taiwan) [28, 35, 39], 2 studies in Australia [24, 29], 7 in the United States [12, 14–16, 19, 22, 34] and the remaining 17 studies in Europe. A total of 6 studies presented sex-specific results, 3 studies were in men [18–20] and 3 in women [15, 16, 30]. There was one study [12] with missing data on age and 4 studies with missing BMI data [12, 21, 33, 36]. The estimated mean age was 57 years (range 34–80 years) and mean value of BMI was 25.4 kg/m^2^ (range 22.3–27.1 kg/m^2^). The duration of follow-up ranged from 5 to 25 years, with a mean follow-up of 13 years. Study characteristics of each dairy intake category by outcomes are shown in Table 2. Results of quality assessment are shown in the Supplemental Table 1, with 18 studies scoring ≥7. All of the studies were funded by a research institute except one study [13] without funding information, thus sub-group analysis was not conducted by funding source. There was no evidence of publication bias in the meta-analyses of milk or dairy consumption with different health outcomes (Supplemental Figs. 19–27).

**Table 1:** Characteristics of 29 prospective cohort studies on dairy consumption and CHD, CVD risk or mortality

References	Study, country	Men (%)	Mean age, year	Mean BMI, kg/m^2^	Follow-up time	No. of cases	No. of subjects	Dairy types included in meta-analysis	Dietary assessment	Outcome; ascertainment	Main confounders
Kahn et al. [12]	California Seventh-Day Adventists, USA	40	–	–	21	6180 deaths	27,530	Milk, Cheese	FFQ (unvalidated)	All-cause mortality; deaths were matched by computer tapes	Age, sex, smoking history, history of major chronic disease
Mann et al. [13]	Vegetarian, semi-vegetarians, and meat eaters; UK	38	34	22.3	13.3	392 deaths (64 fatal IHD)	10,802	Milk, Cheese	FFQ (unvalidated)	All-cause mortality, fatal IHD; National Health Service Central Register, causes of death was coded by investigator blinded	Age, sex, smoking, social class
Appleby et al. [14]	Oxford Vegetarian Study; UK	–	34	22.3	12	63 fatal CHD	10,800	Milk, Cheese	Simple FFQ (unvalidated)	Fatal IHD; National death certificate	Age, sex, smoking, socioeconomic status
Bostick et al. [15]	Postmenopausal women, Iowa; USA	0	61.5	26.8	8	387 fatal IHD	34,486	Total dairy, High-fat dairy	FFQ (validated)	Fata IHD; Registry and follow-up questionnaire	Age, total energy intake, body mass index, waist: ratio, history of diabetes mellitus, cigarette smoking status, postmenopausal oestrogen use, alcohol intake, education, marital status, physical activity, dietary vitamin E and saturated fat intake
Hu et al. [16]	Nurses’ Health Study; USA	0	46.5	24.2	14	939 CHD	41,254	Total dairy, High-fat dairy, Low-fat dairy, Milk	FFQ (validated)	CHD (fatal and nonfatal); medical records reviewed by physicians blind to risk factors; deaths from registry, jps[ota; records or autopsy	Age, time period, BMI, smoking, menopausal status (including hormone replacement therapy), parental history of MI, vitamin E supplement, alcohol, history of hypertension, aspirin, physical activity, total energy intake
Fortes et al. [17]	Elderly residents from public home in Rome; Italy	32	80	25.6	5	53 deaths	162	Cheese	FFQ (validated)	All-cause mortality; Registry	Age, sex, education, BMI, smoking, cognitive function, chronic diseases
Ness et al. [18]	Working men in west of Scotland; UK	100	48	25.3	25	2350 deaths (1212 fatal CVD, 892 fatal CHD)	5765	Milk	Questionnaire (check by interview)	All-cause mortality, fatal CVD, fatal CHD; National Healthy Service Central Registry	Age, smoking, BP, cholesterol, BMI, forced expiratory volume, social class, education, deprivation, siblings, car user, angina, ECG ischemia, bronchitis, alcohol
Al-Delaimy et al. [19]	Health Professionals Follow-up Study	100	53	25.4	12	14,468 IHD (fatal and non-fatal)	39,800	Total dairy, High-fat dairy, Low-fat dairy, Milk	FFQ (validated)	IHD (fatal and nonfatal); medical records reviewed, autopsy reports, death certificates	Age, time period, energy intake, history of diabetes, history of hypercholesterolemia and hypertension, family history of MI, smoking history, aspirin, BMI, alcohol intake, physical activity, vitamin E, trans fatty acids, PUFA:SFA ratio, total protein intake, fibre, folate, n-3 fatty acids, and a-linolenic acid
Elwood et al. [20]	Caerphilly cohort; UK	100	52	26.1	22	811 deaths, 628 CVD, 493 IHD	2512	Milk	FFQ (validated)	All-cause mortality, CVD (fatal and nonfatal), IHD (fatal and nonfatal); ECG examination, GP and hospital records	Age, total energy, smoking, social class, BMI, systolic BP, alcohol and fat, prior vascular disease
Knoops et al. [21]	HALE study (combination of SENECA and FINE studies)	66	75	–	10	1382 deaths	3117	Total dairy	Dietary history	All-cause mortality; general practitioners and/or hospital registers or vital status	Age, sex, alcohol, physical activity, smoking, number of years of education, BMI, chronic diseases, study centre
Paganini-Hill et al. [22]	Leisure World Cohort Study; USA	37	74	23.5	23	11,386 deaths	13,624	Milk	Questionnaire (unvalidated)	All-cause mortality; hospital discharge data, death indexes and ascertainment of death certificates	Age, sex, smoking, exercise, BMI, alcohol, hypertension, angina, MI, stroke, diabetes, rheumatoid arthritis, cancer
Engberink et al. [40]	The Rottedam Study, Netherlands	38	66.9	26.2	11.2	1111 death (307 from CVD)	3971	Total dairy, High-fat dairy, Low-fat dairy, Cheese	FFQ (validated)	All-cause mortality, CVD mortality; medical record and digital record linkage	Age, sex, BMI, SBP, total cholesterol, family history of MI, use of oestrogen, smoking, educational level, alcohol consumption, total energy, saturated fat, intake of fruit, vegetables, meat, fish, coffee, and tea
Panagiotakos et al. [23]	ATTICA Study; Greece	50	53	27	5	30 CVD (fatal and non-fatal)	686	Total dairy, Cheese, Yogurt, Milk	FFQ (validated)	CVD (fatal and non-fatal); medical records	Age, sex, BMI, hypertension, diabetes, hypercholesterolemia, current smoking, physical activity
Bonthuis et al. [24]	Community-based sample, Australia	43	49.8	26.2	14.4	177 death (61 from CVD)	1529	Total dairy, High-fat dairy, Low-fat dairy, Milk, Yogurt, Full-fat cheese	FFQ (validated)	All-cause mortality, CVD mortality; National Death Index of Australia	Age, sex, total energy intake, body mass index, alcohol intake, school leaving age, physical activity level, pack years of smoking, dietary supplement use, b-carotene treatment during trial, presence of any medical condition, and dietary calcium intake.
Goldbohm et al. [25]	Netherland Cohort Study	48	61.6	24.4	10	16,136 death (2689 from IHD)	120,852	Total dairy, High-fat dairy, Low-fat dairy, High-fat fermented dairy, Low-fat fermented dairy, Cheese	150 item FFQ (validated)	All-cause mortality, IHD mortality; Dutch Central Bureau of Genealogy and the Dutch Central Bureau of Statistics	Age, education, cigarette, cigar, and pipe smoking, nonoccupational physical activity, occupational physical activity, BMI, multivitamin use, alcohol, energy, energy-adjusted mono- and polyunsaturated fat intakes, and vegetable and fruit consumption
Sonestedt et al. [26]	The Malmo diet and cancer cohort, Sweden	38	57.3	25.2	12	2520 CVD	26,445	Total dairy, High-fat dairy, Low-fat dairy, Fermented dairy, Milk, Cheese	Dietary assessment method	CVD (fatal and non-fatal)	Sex, season, method, energy intake, BMI, smoking, alcohol consumption, leisure-time physical activity, and education
Dalmeijer et al. [27]	EPIC-NL; Netherlands	25.5	48.7	25.6	10	1184 death, 1807 total CVD, 1309 total CHD,	33,625	Total dairy, High-fat dairy, Low-fat dairy, Fermented dairy, Cheese	79-item FFQ (validated)	All-cause mortality, CVD (fatal and nonfatal), CHD (fatal and nonfatal); Register of hospital discharge diagnoses	Gender, age, total energy intake, physical activity, smoking, education, BMI, ethanol, coffee, fruit, vegetables, fish, meat and bread
Kondo et al. [28]	National Integrated Project for Prospective Observation of Non-communicable Disease And its Trends in the Aged, Japan	44	50.3	22.7	24	893 CVD death, 174 CHD death;	9243	Milk	Weighed diet records and dietary interviews	CVD mortality, CHD mortality; follow-up surveys	Age, body mass index, smoking status, alcohol drinking habit, history of diabetes, use of antihypertensive, work category, and total energy intake
Soedamah-Muthu et al. [31]	Whitehall II Study, United Kingdom	72	56	25.9	10	323 CHD; 237 all-cause mortality	4526	Total dairy, High-fat dairy, Low-fat dairy, Milk, Fermented dairy, Cheese, Yogurt	114 item FFQ (validated)	All-cause mortality, CHD (fatal and non-fatal); Death was collected from NHS Central Registry, cases of MI were identified from twelve-lead electrocardiograms	Age, ethnicity and employment grade, smoking, alcohol intake, BMI, physical activity and family history of CHD/hypertension, fruit and vegetables, bread, meat, fish, coffee, tea and total energy intake
Louie et al. [29]	The Blue Mountain Eye	44	65.4	26.2	15	1048 death	2900	Total dairy,	145-item FF1 (validated)	CVD mortality, CHD mortality;	Age, sex, total energy, baseline
	Study, Australia					(548 from CVD, 432 from CHD)		High-fat dairy, Low-fat dairy		Australian National Death Index	BMI, change in weight during follow up, physical activity level (METs), previous acute myocardial infarction, previous stroke, smoking status, stage II hypertension, type 2 diabetes status, use of antihypertensive medication, use of statins and change in dairy intake
Ruesten et al. [33]	EPIC-Potsdam Study; German	39	50	–	8	363 CVD	23,531	High-fat dairy, Low-fat dairy, High-fat cheese, Low-fat cheese	FFQ (validated)	CVD (fatal and non-fatal); self-administered follow-up questionnaires and medically verified	Age, sex, smoking status, pack-years of smoking, alcohol consumption, leisure-time physical activity, BMI, waist-to-hip ratio, prevalent hypertension at baseline, history of high blood lipid levels at baseline, education, vitamin supplementation and total energy intake
Van Aerde et al. [32]	The Hoorn Study;	43.8	61.1	26.5	12.4	403	1956	Total	92-item FFQ	All-cause	Age, sex, BMI,
	Netherlands					death (116 from CVD, 50 from CHD)		Dairy, High-fat dairy, Low-fat dairy, Milk, Fermented dairy, Cheese	(validated)	Mortality, fatal CVD, fatal CHD; General practitioners and the local hospital	Smoking, educational level, total energy intake, alcohol consumption, physical activity and intake of meat, fish, bread, vegetables, fruit, coffee, and tea
Patterson et al. [30]	Swedish Mammography cohort, Sweden	0	61.2	24.9	11.6	1392 MI	33,636	Total dairy, Milk, Fermented dairy, Low-fat fermented dairy, High-fat fermented dairy, Cheese	96-item semi quantitative FFQ (validated)	Incident cases of MI (fatal and nonfatal); Registry and record linkage	Smoking status, physical activity, waist-to-hip ratio, alcohol consumption, diagnosis of hypertension, diagnosis of high cholesterol, family history of myocardial infarction, education, aspirin usage, hormone therapy usage, energy intake, all other dairy food groups, fruit and vegetables and whole-grain foods, use of oils in cooking, and use of low-fat margarine on bread
Huang et al. [35]	Nutrition and Health Survey in Taiwan, Taiwan	–	35.6	22.9	13.7	444 death (87 from CVD)	3810	Total dairy	FFQ (validated)	All-cause mortality, CVD mortality; National death registration	Age, gender, BMI, region, ethnicity, education level, marriage, history of disease (cardiovascular disease and/or cancer), smoking, drinking, chew betel nut, and supplement use, overall Dietary Index–Revised (dairy score excluded), Calcium intake, vitamin D intake
Haring et al. [34]	Atherosclerosis Risk in Communities Study; USA	44.2	53.8	27.1	22	1147 CHD	12,066	Total dairy, High-fat dairy, Low-fat dairy	FFQ (unvalidated)	CHD (fatal and non-fatal); study visits, yearly telephone follow-up calls, review of hospital discharge lists and medical charts, death certificates, next-of-kin interviews, and physician-completed questionnaires	Age, sex, race, study centre, total energy intake, smoking, education, systolic blood pressure, use of antihypertensive medication, HDL-cholesterol, total cholesterol, use of lipid lowering medication, body mass index, waist-to-hip ratio, alcohol intake, sports-related physical activity, leisure-related physical activity, carbohydrate intake, fibre intake, and magnesium intake
Michaelsson et al. [6]	Swedish Mammography Cohort, Sweden/Cohort of Swedish Men, Sweden	0/100	53.7/60.3	24.7/25.8	20.1/11.2	15,541 death (5278 death from CVD)/10,112 death (4568 death from CVD)	61,433/45,339	Milk, Cheese, Fermented dairy	FFQ (validated)	All-cause mortality and CVD mortality; Swedish cause of death registries	Age, body mass index, height, total energy intake, total alcohol intake, healthy dietary pattern, calcium and vitamin D supplementation, ever use of cortisone, educational level, living alone, physical activity level estimated as metabolic equivalents, smoking status, and Charlson’s comorbidity index; and in women additionally for use of oestrogen replacement therapy and nulliparity
Bergholdt et al. [36]	Copenhagen General Population Study; Denmark	12	56.7	–	5.4	2777 IHD	74,965	Milk	Self-reported questionnaire	IHD (fatal and nonfatal); National DANISH Patient Registry	Age, sex, physical activity in leisure time and at work, smoking, alcohol intake, use of lipid-lowering therapy, fruit, vegetables, fish, fast food, and soda drinks
Praagman et al. [37]	the Rotterdam Study, Netherlands	38	66.9	26.2	17.3	567 CHD (350 fatal)	4235	Total dairy, High-fat dairy, Low-fat dairy, Fermented dairy, Cheese, Yogurt	FFQ (validated)	Total CHD and fatal CHD; medical record and digital record linkage	Age, gender, and total energy intake, BMI, smoking, education level, and alcohol intake, intakes of vegetables, fruit, meat, bread, fish coffee, and tea
Praagman et al. [38]	EPIC-Netherlands cohort	57	48.9	25.6	15	2436 death (727 from CVD, 253 from CHD)	34,409	Fermented dairy, Yogurt, Cheese	FFQ (validated)	All-cause mortality, CVD mortality, CHD mortality; Record linkage and Central Agency for statistics	Age, sex, total energy intake, smoking habit, BMI, physical activity, education level, hypertension at baseline, intakes of alcohol and energy-adjusted intakes of fruit and vegetables
Wang et al. [39]	Japan Collaborative Cohort Study, Japan	42	56.8	22.7 (men); 22.9 (women)	19	21,775 death (6271 death from CVD)	94,980	Milk	Self-administered questionnaires	All-cause mortality and CVD mortality; The date and cause of death were confirmed with the permission of the Director-General of the Prime Minister’s Office	Age categories, smoking status, drinking status, physical activity, sleeping duration, body mass index, education level, participation in health check-ups, green-leafy vegetable intake, and history of hypertension, diabetes, and liver disease

*BMI* body mass index, *CVD* cardiovascular disease, *CHD* coronary heart disease, *FFQ* food frequency questionnaire

**Table 2:** Characteristics and results of linear and nonlinear dose response meta-analyses of dairy exposures

Dairy type (increment g/day)	Outcome	No studies (populations)	Mean age (years)	Mean BMI (kg/m^2^)	median intake range (g/day)	Total N	No events	RR (95% CI)	Heterogeneity I^2^ (%), *P*
Total dairy (per 200 g/day)	Mortality	9 (10)	57.2	25.2	323 (0–713)	175,063	21,222	0.99 (0.96, 1.03)	62.2, 0.005
	CHD	11 (12)	57.4	25.8	360 (20–828)	330,350	8298	0.99 (0.96, 1.02)	38.9, 0.081
	CVD	8	54.4	25.6	339 (0–713)	76,207	5525	0.97 (0.91, 1.02)	59.9, 0.015
High-fat dairy (per 200 g/day)	Mortality	5	56.7	26.0	113 (20–339)	47,126	3407	0.96 (0.88, 1.05)	0.0, 0.603
	CHD	9	55.9	25.9	151 (19–586)	171,627	6661	0.99 (0.93, 1.05)	22.9, 0.240
	CVD	7	57.7	25.9	130 (8–414)	95,242	5408	0.93 (0.84, 1.03)	37.4, 0.143
Low-fat dairy (per 200 g/day)	Mortality	6 (7)	58.5	25.4	217 (0–554)	167,978	19,543	1.01 (0.99, 1.03)	0.0, 0.734
	CHD	9 (10)	55.5	25.7	234 (0–825)	262,228	6244	1.00 (0.97, 1.03)	27.3, 0.193
	CVD	7	57.7	25.9	211 (0–604)	95,242	5408	0.98 (0.95, 1.01)	0.0, 0.769
Milk (per 244 g/day)	Mortality	10 (12)	55.5	24.6	268 (0–878)	268,570	69,355	1.00 (0.93, 1.07)	97.4, <0.001
	CHD	11 (12)	51.1	24.5	227 (0–877)	230,621	8612	1.01 (0.96, 1.06)	45.5, 0.043
	CVD	9 (12)	54.6	24.8	245 (0–878)	249,779	21,580	1.01 (0.93, 1.10)	92.4, <0.001
Fermented dairy (per 20 g/day)	Mortality	11 (19)	57.0	25.2	70 (0–500)	378,058	98,536	0.98 (0.97, 0.99)	94.4, <0.001
	CHD	9 (14)	53.7	25.0	96 (0–417)	256,091	5667	0.99 (0.98, 1.01)	44.6, 0.037
	CVD	9 (17)	54.8	25.8	105 (0–627)	271,071	33,980	0.98 (0.97, 0.99)	87.5, <0.001
Cheese (per 10 g/day)	Mortality	11 (13)	57.2	25.2	25 (1–70)	342,120	54,125	0.99 (0.96, 1.01)	93.3, <0.001
	CHD	9 (10)	53.8	25.0	34 (3–192)	256,091	4022	0.99 (0.97, 1.02)	40.3, 0.089
	CVD	9 (11)	55.3	25.8	34 (0–103)	234,447	15,519	0.98 (0.95, 1.00)	82.6, <0.001
Yogurt (per 50 g/day)	Mortality	3	51.3	25.9	46 (0–145)	40,460	2850	0.97 (0.85, 1.11)	65.8, 0.054
	CHD	3	56.4	25.9	60 (0–145)	98,936	1143	1.03 (0.97, 1.09)	0.0, 0.685
	CVD	3	50.6	26.3	147 (0–627)	36,624	817	1.03 (0.97, 1.09)	0.0, 0.499

### Total, high-fat, and low-fat dairy

Total dairy intake (per 200 g/day) was not associated with the risk of all-cause mortality (Supplemental Figure 1; RR 0.99, 95% CI 0.96–1.03, 10 populations), CHD (Supplemental Figure 2; RR 0.99, 95% CI 0.96–1.02, 12 populations) or CVD (Supplemental Figure 3; RR 0.97, 95% CI 0.91–1.02). Considerable heterogeneity was observed in the meta-analyses of mortality (I^2^ = 62.2%, *P* = 0.005) and CVD (I^2^ = 59.9%, *P* = 0.015) but not CHD (I^2^ = 38.9%, *P* = 0.081). In sensitivity analyses, heterogeneity among studies of the mortality could be reduced to 50% (*P* = 0.042) with a RR of 1.00 (95% CI 0.97–1.04) by excluding the study of Soedamah-Muthu et al. [31]; the heterogeneity among studies of CVD was reduced (I^2^ = 11.2, *P* = 0.338) after removing the study of Hu et al. [16] with a resulting RR of 0.98 (95% CI 0.96–1.00). Sub-group analyses of CHD (Supplemental Table 4) indicated inverse associations for study populations with a mean age >50 years (RR 0.97, 95% CI 0.94–1.00, 8 populations) and also for studies which did not adjust for 7 major confounders defined in methods as age, sex, smoking, alcohol, BMI, physical activity, food energy intake (RR 0.94, 95% CI 0.88–1.00, 3 populations).

High-fat dairy intake (per 200 g/day) showed no association with mortality (Supplemental Figure 4; RR 0.96, 95% CI 0.88–1.05, 5 populations), CHD (Supplemental Figure 5; RR 0.99, 95% CI 0.93–1.05, 9 populations) or CVD (Supplemental Figure 6; RR 0.93, 95% CI 0.84–1.03, 7 populations), and there was no significant heterogeneity. In sensitivity analyses of the association between high-fat dairy and CHD, I-squared was reduced from 22.9% (*P* = 0.240) to 0% (*P* = 0.464) with results of RR 1.01, 95% CI 0.96–1.06) after removing the study of Dalmeijer et al. [27]. Also, sensitivity analyses of the association between high-fat dairy and CVD showed I-squared reduced to 0% (*P* = 0.143) with results of RR 0.98 (95% CI 0.93–1.03) after excluding study Bonthuis et al. [24]. Sub-group analysis of CVD by age showed a stronger inverse association between high-fat dairy intake and CVD risk in the subjects ≤50 years (RR 0.76, 95% CI 0.59–0.97, 3 populations), although the sample size was small. There was no heterogeneity (I^2^ = 31.5%, *P* = 0.232).

Low-fat dairy intake (per 200 g/day) was not significantly associated with mortality (Supplemental Figure 7; RR 1.01, 95% CI 0.99–1.03, 7 populations), CHD (Supplemental Figure 8; RR 1.00, 95% CI 0.97–1.03) or CVD (Supplemental Figure 9; RR 0.98, 95% CI 0.95–1.01). No heterogeneity was found in the meta-analysis on low-fat dairy. In the sub-group analysis for CVD (Supplemental Table 5) on subjects whose BMI > 25 kg/m^2^, low-fat dairy intake was inversely associated with the risk of CVD (RR 0.97, 95% CI 0.94–1.00, 6 populations).

### Milk

Milk intake (per 244 g/day, 12 populations) was not associated with all-cause mortality (Supplemental Figure 10; RR 1.00, 95% CI 0.93–1.07), CHD (Supplemental Figure 11; RR 1.01, 95% CI 0.96–1.06) or CVD (Supplemental Figure 12; RR 1.01, 95% CI 0.93–1.10). Significant heterogeneity was present for all-cause mortality (I^2^ = 97.4, *P* < 0.001), CHD (I^2^ = 45.5, *P* = 0.043) and CVD (I^2^ = 92.4, *P* < 0.001). In sensitivity analyses for the association between milk and all-cause mortality by excluding data of Michaelsson et al. [6] for women, I^2^ reduced to 70.1% (*P* < 0.001) with RR 0.99 (95% CI 0.96–1.01). By removing Kondo et al. [28] from the meta-analysis of CHD, heterogeneity reduced (I^2^ = 35.10, *P* = 0.118) with a RR of 1.01 (95% CI 0.97–1.05). Results of high-fat milk or low-fat milk were not reported, as only one study [30] was available for the effect of high-fat milk or low-fat milk in relation to CHD. Sub-group analyses showed an inverse association between milk consumption and mortality (Supplemental Table 3) in the subgroup of studies with a mean age ≤50 years (3 populations without heterogeneity (I^2^ = 0%, *P* = 0.479). Also, inverse associations were found between milk intake and CVD (Supplemental Table 5) for the studies which did not adjust for 7 confounders (age, sex, smoking, alcohol, BMI, physical activity, food energy intake) (RR 0.94, 95% CI 0.89–0.99; I^2^ = 28.6, *P* = 0.210) or for the NOS score <7 (RR 0.95, 95% CI 0.90–1.00; I^2^ = 22.1, *P* = 0.278).

### Total fermented dairy, cheese and yogurt

**Fig. 2:**
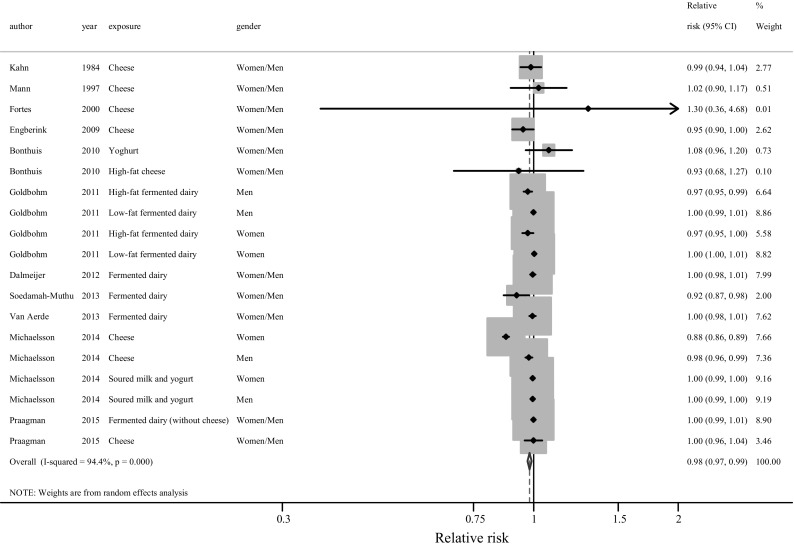
Relative risk of all-cause mortality for an increment of 20 g/day of fermented dairy intake. *Squares* represent study-specific RR. *Square areas* are proportional to the overall specific-study weight to the overall meta-analysis. *Horizontal lines* represent 95% CIs. *Diamonds* represent the pooled relative risk and 95% CIs. By excluding the Swedish study [6] of women’s results for cheese, RR = 1.00 (95% CI 0.99–1.00), I^2^ = 45.2% (*P* = 0.02)

**Fig. 3:**
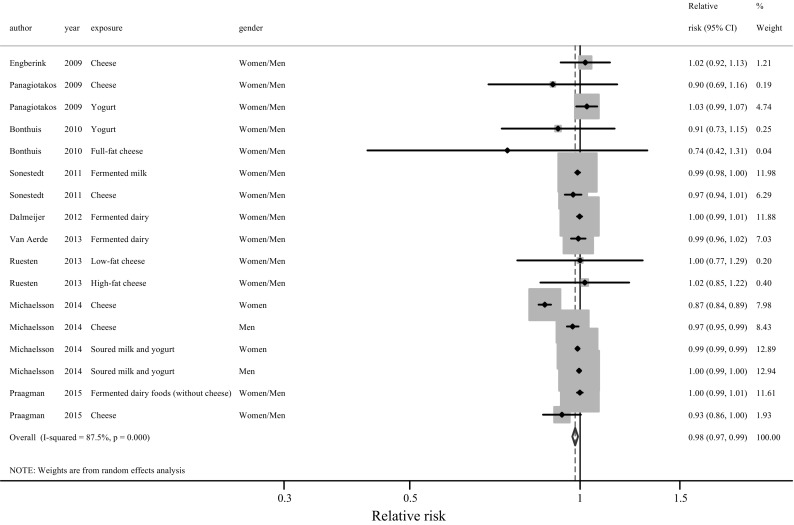
Relative risk of CVD for an increment of 20 g/day of fermented dairy intake. *Squares* represent study-specific RR. *Square areas* are proportional to the overall specific-study weight to the overall meta-analysis. *Horizontal lines* represent 95% Cis. *Diamonds* represent the pooled relative risk and 95% CIs. By excluding the Swedish study [6] of women’s results for cheese, RR = 0.99 (95% CI 0.99–1.00), I^2^ = 23.8% (*P* = 0.19)

Total fermented dairy intake (weighted median intake 77 g/day, 19 populations, 11 studies) was non-linearly and marginally associated with lower mortality risk, with a RR of 0.98 (95% CI 0.97–0.99) per 20 g/day but with high heterogeneity (I^2^ = 94.4%, *P* < 0.001; Fig. 2). In sensitivity analysis, by excluding the Swedish study [6] of women’s results for cheese, I^2^ was reduced to 45.2% (*P* = 0.02), with RR of 1.00 (95% CI 0.99–1.00). Similarly, total fermented dairy intake (17 populations, 9 studies) was non-linearly and modestly associated with a 2% lower CVD risk per 20 g/day (RR 0.98, 95% CI 0.97–0.99) (Fig. 3). Significant heterogeneity was present (I^2^ = 87.5%, *P* < 0.001). Again, in a sensitivity test, excluding the Swedish study [6] of women’s results for cheese, showed a marked decrease in heterogeneity to 23.8% (*P* = 0.19), with a 1% lower CVD risk (RR 0.99, 95% CI 0.99–1.00). Total fermented dairy intake (14 populations, 9 studies) showed no association with CHD risk, with a RR of 0.99 (95% CI 0.98–1.01) per 20 g/day increment with no indications of a nonlinear association (Supplementary Figure 13). The heterogeneity in the CHD and total fermented dairy data was significant (I^2^ = 44.6%, *P* = 0.037). In sensitivity analyses, after excluding the study of Patterson et al. [30], the heterogeneity for cheese was reduced (I^2^ = 32.5%; *P* = 0.122), but with results remaining similar with a RR of 1.00 (95% CI 0.99–1.01).

**Fig. 4:**
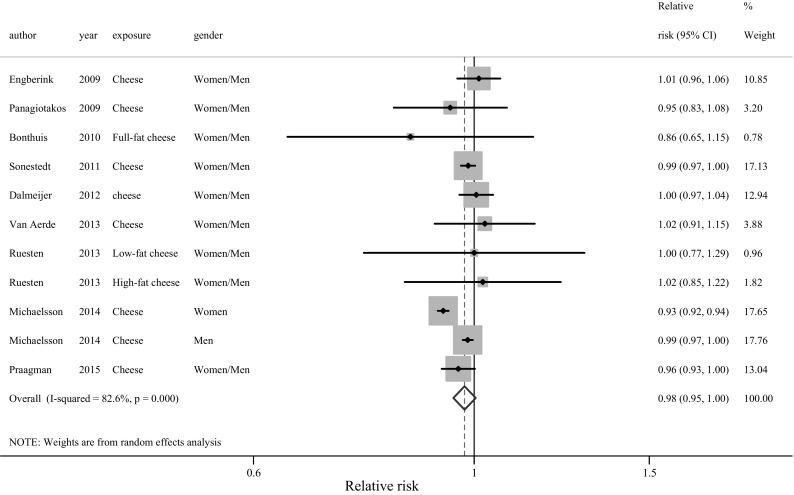
Relative risks of CVD for an increment of 10 g/day of cheese. *Squares* represent study-specific RR. *Square areas* are proportional to the overall specific-study weight to the overall meta-analysis. *Horizontal lines* represent 95% CIs. *Diamonds* represent the pooled relative risk and 95% Cis. By excluding the Swedish study [6] of women’s results for cheese, RR = 0.99 (0.98–0.99), I^2^ = 0% (*P* = 0.84)

Cheese (per 10 g/day) was marginally non-linearly inversely related to CVD (Fig. 4; RR 0.98, 95% CI 0.95–1.00; 11 populations), but not to risk of mortality (Supplementary Figure 14; RR 0.99, 95% CI 0.96–1.01; 13 populations) or CHD (Supplementary Figure 15; RR 0.99, 95% CI 0.97–1.02). Significant heterogeneity was seen for mortality (I^2^ = 93.3%, *P* < 0.001) or CVD (I^2^ = 82.6%, *P* < 0.001). In sensitivity analyses, heterogeneity was reduced after removal of the large Swedish study [6] (I^2^ = 11%, *P* = 0.337 for mortality; I^2^ = 0%, *P* = 0.835 for CVD), with no association for mortality and CVD (RR = 1 for both).

Yogurt (3 populations) was not associated with all-cause mortality (I^2^ = 65.8%, *P* = 0.054, RR 0.97, 95% CI 0.85–1.11), CHD (I^2^ = 0%, *P* = 0.685, RR 1.03, 95% CI 0.97–1.09) or CVD (I^2^ = 0%, *P* = 0.499, RR 1.03, 95% CI 0.97–1.09) (Supplementary Figure 16–18).

## Discussion

This meta-analysis combining data from 29 prospective cohort studies showed there were no associations between total dairy, high- and low-fat dairy, milk and the health outcomes including all-cause mortality, CHD or CVD. The modest inverse associations of total fermented dairy were found with all-cause mortality and CVD, but not CHD. By examining different types of fermented food in relation to CVD, we found marginally inverse association with cheese but not yogurt. However, further sensitivity tests showed the inverse associations of fermented dairy and cheese with all-cause mortality or CVD disappeared after removing the study of Michaelsson et al. [6].

No associations were found between total dairy and milk consumption with all-cause mortality, CHD or CVD in the current study, which is in agreement with several meta-analyses [47, 48]. Larsson et al. [47] reported neutral associations of dairy and milk consumption with mortality or CVD mortality. Mullie et al. [48] reported neutral associations of milk consumption with all-cause mortality or CHD. In addition, the current study is in agreement with a recently published review [49] which indicated neutral associations between the consumption of total dairy and risk of CHD or CVD. Results of sub-group analyses showed the inverse associations were observed between total dairy intake and CHD, or the association between milk consumption and CVD when studies did not adjust for major confounders. Thus, confounders included in statistical analyses in prospective studies have substantial effects on the final findings and conclusions. Furthermore, inverse associations were also found in sub-groups of studies defined by mean age (≤50, >50 years) or BMI (>25 kg/m^2^) of the associations between total, high-fat, low-fat dairy and milk with risk of all-cause mortality, CHD or CVD, which indicated the findings and conclusions were also affected by characteristics of the study populations within different studies.

Three US prospective cohort studies described by Chen et al. [50] showed a substantially lower risk of CVD when animal fats, including dairy fat, were replaced by unsaturated fats. Recently, UK National Health Service (NHS) has recommended low-fat milk and dairy products as healthy choices [51]. However, in the current study, high-fat and low-fat dairy consumption were investigated separately and no substitution models replacing high by low-fat dairy products were carried out. We found no significant associations between high-and low-fat dairy and all-cause mortality, CHD or CVD. This supports two previous meta-analyses [5, 52] which also reported no association of high or low-fat dairy and CHD. Furthermore, beneficial effects of high-fat dairy foods on human health were reported by a cross-sectional study [53], which showed an inverse association of full-fat dairy food and the metabolic syndrome. In addition, another US study [54], which reviewed cross-sectional and prospective cohort studies, showed that 11 of the 16 studies identified that population with higher full-fat dairy intake had less adiposity. It is also noteworthy that butter as a high fat dairy food containing 80% fat [55], a recent meta-analysis on the effects of butter [10] showed that whilst consumption was weakly associated with all-cause mortality (per 14 g/day: RR 1.01, 95% CI 1.00–1.03), there was no significant association with CHD, CVD or stroke and there was an inverse association with incidence of diabetes (RR 0.96, 95% CI 0.93–0.99). Therefore, the effect of dairy fat on CVD is complex and may be influenced by the nature of the fat containing food vehicle, which needs confirmation in further studies.

Despite their fat content and composition, milk and dairy products are naturally rich in various minerals (e.g. calcium, potassium), protein and vitamins (e.g. vitamin A and vitamin B_12_) [56]. Nutrients including calcium, potassium and magnesium have been suggested to be associated with lower risk of stroke [57, 58]. Short-term human intervention studies [59, 60] also indicated that subjects who have high-fat diets enriched with dairy minerals or calcium have significantly lower total cholesterol and LDL-cholesterol levels than those on a control diet. This may explain in part why total dairy consumption has a neutral role in terms of the effect on health outcomes.

The current study also showed total fermented dairy and cheese intake to be marginally inversely associated with mortality and CVD risk, respectively, and large heterogeneity was present. However, by removing the study of Michaelsson et al. [6], heterogeneity of the associations of total fermented dairy and mortality or CVD, cheese and mortality or CVD were markedly reduced. Also, the marginally inversely associations were disappeared. To our knowledge, the present study is the first dairy meta-analysis to include the large Swedish cohort results [6]. The markedly reduced heterogeneity after removing the results of the Swedish female cohort [6] indicated the heterogeneous nature of the Swedish study, which may be related to the diet and lifestyle characteristics of the study participants, as they had a relatively low education level (80 and 70% for women and men were educated for ≤9 years, respectively), also the highest milk drinkers had highest percentage of smokers and those living alone.

Cheese consumption based on 11 populations was found to be modestly and inversely associated with CVD risk, with a 2% lower risk of CVD per 10 g/day of cheese, however, the significant association disappeared after removing the study of Michaelsson et al. [6]. Compared with other meta-analyses on cheese, Alexander et al. [4] has reported 11% lower risk of CVD per 35 g/day (95% CI 0.78–1.01), while Chen et al. [61] presented 10% lower risk of CHD per 50 g/day (95% CI 0.84–0.95). However, the analysis of the associations between cheese and CVD in studies of Alexander et al. [4] and Chen et al. [61] were based on 3 and 8 populations, respectively, which was less than our current study of 11 populations.

Furthermore, total fermented dairy and cheese were modestly inversely associated with risk of CVD but not CHD in the current meta-analysis, so perhaps both dairy types play a role in reducing the risk of stroke. This is supported by the evidence of another recent meta-analysis [9], which found a 9% lower risk of stroke (RR 0.91, 95% CI 0.82–1.01) associated with higher total fermented dairy intake and a 3% lower risk of stroke (RR 0.97, 95% CI 0.94–1.01) with higher cheese consumption, although none of these associations were statistically significant. As there was limited information of the different sub-types of the CVD events, the understanding of the association of fermented dairy products with varied CVD types remains unclear. In addition, unlike the result for cheese, the association of yogurt with disease outcomes was neutral. However, a previous review of randomised trials suggested that yogurt is associated with lower risk of CVD [62]. Our null results for yogurt intake and CVD may be due to the limited number of participants from only 3 populations. In addition, a very recent meta-analysis showed a 14% lower risk of type 2 diabetes for 80 g/day yogurt intake (RR 0.86, 95% CI 0.83–0.90) based on 11 prospective cohort studies [63].

The mechanism of the beneficial association of fermented dairy products and reduced CVD risk and mortality is uncertain. Evidence from randomised controlled trials suggests that the reason, at least in part, may be an effect of the food matrix reducing lipid absorption and short chain fatty acids produced by the bacteria in the large intestine [64]. Moreover, omics-techniques have suggested that some of the beneficial effects of cheese can be accounted for by microbial fermentation producing short chain fatty acids such as butyrate [65].

Strengths of our study include the use of dose–response meta-analysis, the inclusion of more studies than in previous meta-analyses and the consideration of examination the individual dairy products separately such as dairy products in terms of fat content (high-fat, low-fat) or processing method (fermented or non-fermented). However, investigation of total dairy or total fermented dairy consumption with disease outcomes by combining dairy foods, high and low-fat dairy foods, solids and liquids, simply adding these up is a limitation which should be addressed in future studies by collecting and analyzing more detailed data. In addition, limitations of the study include sub-group analyses that lack statistical power, such as for Asian studies and effects of gender. We have 9 studies with scores of 7 or less by using the Newcastle–Ottawa Scale (NOS) [43]. Study quality could explain some heterogeneity but not all. For example, NOS scores of all studies containing high-fat dairy or low-fat dairy were ≥7, which could have resulted in lower heterogeneity for those analyses. Furthermore, residual confounding is a limitation of prospective cohort studies. The background diet should be taken into account in the statistical analyses as major confounders, which was done in 15 out of 29 cohort studies. Comparisons of dairy products with other foods in replacement models were not possible from the available data. The neutral risks of dairy products with mortality and CVD risk could be because of replacement by other foods, for example, those with high intake of dairy products may consume less sugar sweetened beverages which could lead to lower CVD mortality [66] or consume more processed meat which could lead to higher CVD risks [67, 68]. For future studies it is important to investigate in more detail how dairy products can be replaced by other foods.

## Conclusions

The current meta-analysis of 29 prospective cohort studies suggested neutral associations of total, high and low-fat dairy, milk and yogurt with risk of all-cause mortality, CHD and CVD. In addition, a possible role of fermented dairy was found in CVD prevention, but the result was driven by a single study.

## Electronic supplementary material

Below is the link to the electronic supplementary material.

Supplementary material 1 (DOCX 345 kb)

Supplementary material 2 (DOCX 19 kb)
