# Supplementary material for: Milk and dairy consumption and risk of cardiovascular diseases and all-cause mortality: dose–response meta-analysis of prospective cohort studies
Source: Eur J Epidemiol. 2017 Apr 3;32(4):269–87. doi: 10.1007/s10654-017-0243-1 (PMC5437143; doi:10.1007/s10654-017-0243-1)
Supplement: Supplementary file 1 — Supplementary material 1 (DOCX 345 kb) [file 10654_2017_243_MOESM1_ESM.docx]

**Milk and dairy consumption and risk of cardiovascular diseases and all-cause mortality: dose-response meta-analysis of prospective cohort studies.**

Jing Guo^1^, Arne Astrup^2^, Julie A. Lovegrove^3^, Lieke Gijsbers^4^, David I Givens^1^, Sabita S. Soedamah-Muthu^4^

^1^ Centre for Food, Nutrition and Health, University of Reading, RG6 6AR, UK, JG: PhD, jing.guo@pgr.reading.ac.uk. DIG: Professor of Food Chain Nutrition and Head of Centre for Food, Nutrition and Health, d.i.givens@reading.ac.uk.

^2^ Department of Nutrition, Exercise and Sports, University of Copenhagen, DK-2200, Denmark, Professor and Head of Department of Nutrition, Exercise and Sports, ast@nexs.ku.dk.

^3^ Hugh Sinclair Unit of Human Nutrition and Institute for Cardiovascular and Metabolic Research, University of Reading, RG6 6AP, UK, Professor and Hugh Sinclair Chair in Human Nutrition, j.a.lovegrove@reading.ac.uk.

^4^ Division of Human Nutrition, Wageningen University and Research, Wageningen, 6708 WE, the Netherlands. LG: PhD, lieke.gijsbers@wur.nl. SSSM: Assistant Professor, sabita.soedamah-muthu@wur.nl.

Correspondence to: Jing Guo^1^, [jing.guo@pgr.reading.ac.uk](mailto:jing.guo@pgr.reading.ac.uk)

**Milk and dairy consumption and risk of cardiovascular diseases and mortality: dose-response meta-analysis of prospective cohort studies.**

CONTENT

Supplemental Methods

- Search strategy.
- Newcastle-OTTAWA quality assessment scale to determine quality of prospective cohort studies.

Supplemental Tables

- Supplemental Table 1. Quality assessment of prospective cohort studies on dairy intake, risk of CHD, CVD and all-cause mortality.
- Supplemental Table 2. Definition of dairy products as described in original 29 prospective cohort studies included in the meta-analysis.
- Supplemental Table 3. Association between dairy foods and all-cause mortality by subgroups.
- Supplemental Table 4. Association between dairy foods and CHD by subgroups.
- Supplemental Table 5. Association between dairy foods and CVD by subgroups

Supplemental Figures

- Supplemental Figure 1. Forest plot for the association between total dairy intake and all-cause mortality.
- Supplemental Figure 2. Forest plot for the association between total dairy intake and CHD.
- Supplemental Figure 3. Forest plot for the association between total dairy intake and CVD.
- Supplemental Figure 4. Forest plot for the association between high-fat dairy intake and all-cause mortality.
- Supplemental Figure 5. Forest plot for the association between high-fat dairy intake and CHD.
- Supplemental Figure 6. Forest plot for the association between high-fat dairy intake and CVD.
- Supplemental Figure 7. Forest plot for the association between low-fat dairy intake and all-cause mortality.
- Supplemental Figure 8. Forest plot for the association between low-fat dairy intake and CHD.
- Supplemental Figure 9. Forest plot for the association between low-fat dairy intake and CVD.
- Supplemental Figure 10. Forest plot for the association between milk intake and all-cause mortality.
- Supplemental Figure 11. Forest plot for the association between milk intake and CHD.
- Supplemental Figure 12. Forest plot for the association between milk intake and CVD.
- Supplemental Figure 13. Forest plot for the association between total fermented dairy intake and CHD.
- Supplemental Figure 14. Forest plot for the association between cheese intake and all-cause mortality.
- Supplemental Figure 15. Forest plot for the association between cheese intake and CHD.
- Supplemental Figure 16. Forest plot for the association between yogurt intake and all-cause mortality.
- Supplemental Figure 17. Forest plot for the association between yogurt intake and CHD.
- Supplemental Figure 18. Forest plot for the association between yogurt intake and CVD.
- Supplemental Figure 19. Funnel plot for studies of the association between total dairy intake and all-cause mortality.
- Supplemental Figure 20. Funnel plot for studies of the association between total dairy intake and CHD.
- Supplemental Figure 21. Funnel plot for studies of the association between low-fat dairy intake and CHD.
- Supplemental Figure 22. Funnel plot for studies of the association between milk intake and all-cause mortality.
- Supplemental Figure 23. Funnel plot for studies of the association between milk intake and CHD.
- Supplemental Figure 24. Funnel plot for studies of the association between milk intake and CVD.
- Supplemental Figure 25. Funnel plot for studies of the association between fermented dairy intake and CHD.
- Supplemental Figure 26. Funnel plot for studies of the association between cheese intake and all-cause mortality.
- Supplemental Figure 27. Funnel plot for studies of the association between cheese intake and CHD.
- Supplemental Figure 28. Ding’s Spaghetti plot for the association between total dairy intake and all-cause mortality.

**Supplemental Methods**

Search strategy (PubMed) – updated until Sep 2016

EMBASE (<http://www.embase.com>) and SCOPUS (<http://www.scopus.com>) search strategies were based on the PubMed (<http://www.ncbi.nlm.nih.gov/pubmed>) query syntax which is shown below.

PubMed (<http://www.ncbi.nlm.nih.gov/pubmed>)

Action 1 Determinants

#1 dairy [Title/Abstract]) OR milk*[Title/Abstract]) OR cheese*[Title/Abstract]) OR yogurt*[Title/Abstract]) OR yogurt*[Title/Abstract]) OR butter [Title/Abstract]) OR buttermilk [Title/Abstract]) OR dietary pattern*[Title/Abstract]

#2 dairy products [MeSH Terms]) OR milk [MeSH Terms]) OR cheese [MeSH Terms]) OR yogurt[MeSH Terms]) OR butter[MeSH Terms]) OR cultured milk products[MeSH Terms]

#3 custard*[Title/Abstract]) OR pudding*[Title/Abstract]) OR cream*[Title/Abstract]) OR cream[Title/Abstract]) OR ice cream[Title/Abstract]) OR ice-cream[Title/Abstract]) OR curd*[Title/Abstract]) OR porridge[Title/Abstract]

#4 diet[Title/Abstract]) OR diets[Title/Abstract]) OR dietary[Title/Abstract]) OR intake*[Title/Abstract]) OR suppl*[Title/Abstract]) OR consumption[Title/Abstract]) OR food*[Title/Abstract]) OR drink*[Title/Abstract]) OR meal[Title/Abstract]) OR nutrition[Title/Abstract]) OR nutrient*[Title/Abstract]) OR products[Title/Abstract]

#5 [(#1 OR #2 OR #3) AND #4]

Action 2 Outcome

#6 diet[Title/Abstract]) OR diets[Title/Abstract]) OR dietary[Title/Abstract]) OR intake*[Title/Abstract]) OR suppl*[Title/Abstract]) OR consumption[Title/Abstract]) OR food*[Title/Abstract]) OR drink*[Title/Abstract]) OR meal[Title/Abstract]) OR nutrition[Title/Abstract]) OR nutrient*[Title/Abstract]) OR products[Title/Abstract]

#7 cardiovascular [Title/Abstract]) OR vascular [Title/Abstract]) OR CVD [Title/Abstract]) OR Cardiovascular Diseases [Mesh:NoExp]

#8 coronary[Title/Abstract]) OR cardiac[Title/Abstract]) OR heart[Title/Abstract]) OR infarction*[Title/Abstract]) OR infarct*[Title/Abstract]) OR ischaemic[Title/Abstract]) OR ischemic[Title/Abstract]) OR ischaemia[Title/Abstract]) OR ischemia[Title/Abstract]) OR CHD[Title/Abstract]) OR CAD[Title/Abstract]) OR MI[Title/Abstract]) OR myocard*[Title/Abstract]) OR Coronary Artery Disease[Mesh:NoExp]) OR coronary disease[Mesh:NoExp]

#9 cerebrovascular*[Title/Abstract]) OR stroke[Title/Abstract]) OR CVA[Title/Abstract]) OR Cerebrovascular disease[Mesh:NoExp]) OR stroke[Mesh:NoExp]

#10 (#6 OR #7 OR #8 OR #9)

Action 3 Combine exposure and outcome

#11 (#5 AND #10)

Action 4 Limits

#12 Rats[Mesh:NoExp]) OR Mice[Mesh:NoExp]) OR rat[Title/Abstract]) OR rats[Title/Abstract]) OR mouse[Title/Abstract]) OR mice[Title/Abstract]) OR vivo[Title/Abstract]) OR vitro[Title/Abstract])

#13 (#11 NOT #12)

**NEWCASTLE – OTTAWA QUALITY ASSESSMENT SCALE**

**COHORT STUDIES**

Note: A study can be awarded a maximum of one star for each numbered item within the Selection and Outcome categories. A maximum of two stars can be given for Comparability.

**Selection**

1) Representativeness of the exposed cohort

a) truly representative of the average *healthy adults* in the community
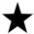


b) somewhat representative of the average *healthy adults* in the community
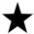


c) selected group of users *e.g. nurses, volunteers, vegetarian*

d) no description of the derivation of the cohort

2) Selection of the non-exposed cohort

a) drawn from the same community as the exposed cohort
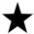


b) drawn from a different source

c) no description of the derivation of the non-exposed cohort

3) Ascertainment of exposure

a) secure record (*e.g. 7 day food diary)*
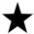


b) structured interview/≥ 2 *dietary recalls/diet history/ food frequency questionnaire validated for dairy components*
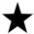


c) written self-report (*e.g. <2 dietary recalls/non-validated food frequency questionnaire or not reported whether food frequency questionnaire was validated*)

d) no description

4) Demonstration that outcome of interest was not present at start of study

a) yes
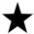


b) no

**Comparability**

1) Comparability of cohorts on the basis of the design or analysis

a) study controls for *age, sex, smoking, total energy intake, and body mass index*
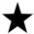


b) study controls for any additional factor (*e.g. physical activity, alcohol intake, family history of diabetes, dietary factors*)
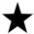


**Outcome**

1) Assessment of outcome

a) independent blind assessment (e.g. clinical diagnosis/complete medical information available).
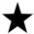


b) record linkage/*medical record or validated self-report*
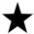


c) non-validated self-report

d) no description
2) Was follow-up long enough for outcomes to occur

a) yes/ *follow up period for outcome of interest is 10 years or over*
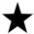


b) no

3) Adequacy of follow-up of cohorts

a) complete follow-up - all subjects accounted for
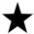


b) subjects lost to follow-up unlikely to introduce bias - small number lost ≤*20%* follow-up, or description provided of those lost
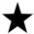


c) follow-up rate <*80%* or no description of those lost

d) no statement

Supplemental Table 1. Quality assessment of cohorts studies on dairy intake, risk of CHD, CVD or all-cause mortality.

|  | **Selection** | | | | **Comparability** | **Outcome** | | | **Total score** |
| --- | --- | --- | --- | --- | --- | --- | --- | --- | --- |
|  | **Representativeness of the exposed cohort** | **Selection of the non-exposed cohort** | **Ascertainment of exposure** | **Outcome not present at start of study** | **Comparability of cohorts on the basis of the design or analysis** | **Assessment of outcome** | **Follow-up long enough for outcomes to occur** | **Adequacy of follow-up of cohorts** |  |
| Kahn et al, 1984 [1] | B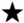 | A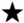 | C | B | - | B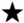 | A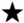 | B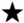 | 5 |
| Mann et al, 1997 [2] | C | A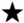 | C | A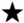 | - | B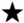 | A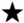 | D | 4 |
| Hu et al, 1999 [3] | C | A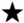 | B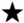 | A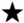 | A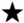 B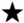 | B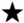 | A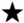 | B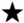 | 8 |
| Appleby et al, 1999 [4] | C | A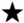 | B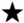 | B | - | B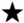 | A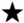 | B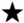 | 6 |
| Bostick et al, 1999 [5] | C | A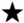 | B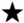 | A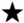 | A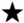 B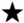 | B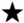 | B | B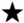 | 9 |
| Fortes et al, 2000 [6] | C | A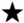 | B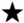 | A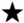 | - | B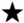 | B | B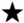 | 6 |
| Ness et al, 2001 [7] | B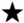 | A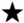 | D | A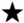 | - | B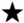 | A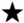 | D | 5 |
| Al-Delaimy et al, 2003 [8] | C | A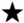 | B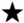 | A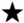 | A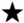 B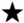 | B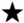 | A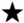 | B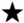 | 8 |
| Elwood et al, 2004 [9] | B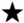 | A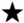 | A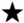 | A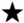 | A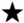 B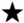 | A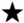 | A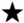 | B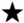 | 8 |
| Knoops et al, 2006 [10] | B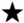 | A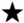 | B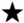 | A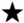 | - | D | A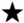 | D | 5 |
| Paganini-Hill et al, 2007 [11] | B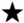 | A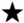 | C | A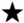 | - | B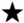 | A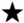 | B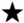 | 6 |
| Panagiotakos et al, 2009 [12] | B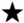 | A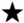 | B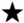 | A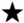 | - | B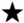 | B | C | 5 |
| Engberink et al, 2009 [13] | A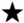 | A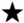 | B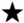 | A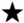 | A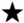 B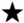 | A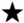 | A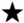 | D | 8 |
| Bonthuis et al. 2010 [14] | B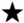 | A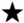 | B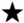 | A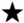 | A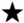 B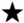 | B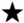 | A | B | 8 |
| Goldbohm et al. 2011 [15] | A | A | B | A | A B | B | A | B | 9 |
| Sonestedt et al. 2011 [16] | B | A | A | A | A B | A | A | A | 9 |
| Kondo et al. 2012 [17] | A | A | B | A | A B | B | A | B | 9 |
| Dalmeijer et al, 2012 [18] | A | A | B | A | A B | B | A | B | 9 |
| Patterson et al. 2013 [19] | B | A | B | A | - | B | A | C | 6 |
| Soedamah-Muthu et al. 2013 [20] | C | A | B | A | A B | A | A | D | 7 |
| Louie et al. 2013 [21] | C | A | B | A | A B | B | A | B | 9 |
| von Ruesten et al, 2013 [22] | A | A | B | A | A B | B | B | A | 8 |
| Van Aerde et al, 2013 [23] | B | A | B | A | A B | B | A | B | 9 |
| Michaelsson et al. 2014 [24] |  | | | | | | | | |
| -Swedish Mammography Cohort | B | A | A | A | A B | B | A | C | 8 |
| -The Cohort of Swedish Men | B | A | A | A | A B | B | A | C | 8 |
| Praagman et al. 2014 (Rotterdam) [25] | A | A | B | A | A B | A | A | B | 8 |
| Haring et al, 2014 [26] | B | A | C | A | A B | B | A | C | 7 |
| Huang et al. 2015 [27] | A | A | B | A | - | B | A | D | 6 |
| Bergholdt et al, 2015 [28] | A | A | B | A | - | A | B | B | 6 |
| Praagman et al. 2015 [29] | B | A | B | A | A B | A | A | B | 9 |
| Wang et al. 2015 [30] | B | A | C | A | - | B | A | A | 6 |

Supplemental Table 2. Definition of dairy products as described in the paper of 29 prospective cohort studies included in the meta-analysis.

| Exposure category in original paper | Exposure category  in meta-analysis | Definition (if available) |
| --- | --- | --- |
| *Kahn et al, 1984* [1] |  |  |
| Milk | Milk | Not further defined |
| Cheese | Cheese | Not further defined |
| *Mann et al, 1997* [2] |  |  |
| Milk | Milk | Not further defined |
| Cheese | Cheese | Cheese (excluding cottage) |
| *Hu et al, 1999* [3] |  |  |
| Total dairy | Total dairy | Not further defined |
| High-fat dairy | High-fat dairy | Whole milk, hard or cream cheese, ice cream, and butter |
| Low-fat dairy | Low-fat dairy | Skim or low-fat milk, yogurt, and cottage cheese |
| Milk | Milk | Not further defined |
| *Appleby et al, 1999* [4] |  |  |
| Milk | Milk | Not further defined |
| Cheese | Cheese | Not further defined |
| *Bostick et al, 1999* [5] |  |  |
| Total dairy | Total dairy | Milk products excluding butter |
| Fat-containing dairy intake | High-fat dairy | Milk products other than butter containing fat (exclude skim milk) |
| *Fortes et al, 2000* [6] |  |  |
| Cheese | Cheese | Not further defined |
| *Ness et al, 2001* [7] |  |  |
| Milk | Milk | Milk |
| *Al-Delaimy et al, 2003* [8] |  |  |
| Total dairy | Total dairy | Not further defined |
| High-fat dairy | High-fat dairy | Not further defined |
| Low-fat dairy | Low-fat dairy | Not further defined |
| Milk | Milk | Not further defined |
| *Elwood et al, 2004* [9] |  |  |
| Milk | Milk | Liquid milk, not milk used in food preparation |
| *Knoops et al, 2006* [10] |  |  |
| Milk and milk products | Total dairy | Not further defined |
| *Paganini-Hill et al, 2007* [11] |  |  |
| Milk | Milk | Milk |
| *Panagiotakos et al, 2009* [12] |  |  |
| Dairy products | Total dairy | Not further defined |
| Cheese | Cheese | Not further defined |
| Yogurt | Yogurt | Not further defined |
| Milk | Milk | Not further defined |
| *Bonthuis et al. 2010* [14] |  |  |
| Total dairy | Total dairy | Skim milk, low-fat milk, low-fat yogurt, cottage or ricotta cheese, whole milk, cream, ice cream, yogurt, full-fat cheese and custard |
| Low-fat dairy | Low-fat dairy | Skim milk, low-fat milk, low-fat yogurt, cottage or ricotta cheese |
| Full-fat dairy | Full-fat dairy | Whole milk, cream, ice cream, yogurt, full-fat cheese and custard |
| Milk | Milk | Whole milk, skimmed and low-fat milk |
| Yogurt | Yogurt | Not further defined |
| Full-fat cheese | Full-fat cheese | Not further defined |
| *Goldbohm et al. 2011* [15] |  |  |
| Milk products | Total dairy | Milk, yogurt, buttermilk, quark, and dishes in which these foods were used |
| Nonfermented full-fat milk | High-fat dairy | Whole milk (3.7% fat), cream (36%, 20% fat), condensed whole milk, whole-milk cocoa, pudding, and ice cream |
| Nonfermented low-fat milk | Low-fat dairy | Low-fat milk (1.5% fat), skim milk (0.1% fat), condensed low-fat milk, and low-fat and skim cocoa |
| Fermented full-fat milk | High-fat fermented dairy | Yogurt (3.5% fat), full-fat quark (fresh cheese), and sour cream |
| Fermented low-fat milk | Low-fat fermented dairy | Buttermilk, skim yogurt (0.1% fat), and non-fat quark (fresh cheese) |
| Cheese | Cheese | Not further defined |
| butter | Butter | Not further defined |
| Low-fat dairy | Low-fat dairy | Not further defined |
| *Sonestedt et al. 2011* [16] |  |  |
| Total dairy | Total dairy | Milk, cheese (>10% fat), cream, butter (including the milk-based spread Bregott) |
| Milk | Milk products | Fermented (yogurt and processed sour milk), non-fermented milk products |
| Fermented milk | Fermented dairy | Yogurt and processed sour milk |
| Low-fat milk | Low-fat dairy | Milk and milk products≤2.4% fat |
| high-fat milk | High-fat dairy | Milk and milk products>2.4% fat |
| cheese | Cheese | Cheese>10% fat |
| *Kondo et al. 2012* [17] |  |  |
| Milk and dairy product consumption | Milk | 93% was in the form of milk |
| Dalmeijer et al, 2012 [18] |  |  |
| Total dairy intake | Total dairy intake | All dairy food products except for butter and ice cream. |
| High-fat dairy | High-fat dairy | Milk and milk products with a fat content ≥2 g/100 g (whole milk products) or cheese products with a fat content ≥20 g/100 g. |
| Low-fat dairy | Low-fat dairy | Milk and milk products with a fat content<2 g/100 g (skimmed or semi-skimmed milk products) or cheese with a fat content< 20 g/100 g |
| Cheese | Cheese | All types of cheese except for curd |
| Fermented dairy | Fermented dairy | Buttermilk, yogurts, and cheese |
| *Patterson et al. 2013* [19] |  |  |
| Total dairy foods | Total dairy | Total dairy intake was the sum of milk [full-fat (≥3.0% fat), semi-skimmed (≤1.5% fat), skimmed (0.5% fat), and pancakes], cultured milk/yogurt [full-fat (≥3.0% fat) and low-fat (≤1.5% fat)], cheese [full-fat (>17% fat), low-fat (≤17% fat), and cottage cheese/quark], cream and crème fraiche (full-fat and low-fat) intakes. |
| Milk | Milk | Full-fat (≥3.0% fat), semi-skimmed (≤1.5% fat), skimmed (0.5% fat), and pancakes (A serving of pancakes contributed one serving of total milk) |
| Low-fat milk | Low-fat milk | Semi-skimmed (≤1.5% fat) and skimmed (0.5% fat) |
| Full-fat milk | Full-fat milk | Milk (≥3.0% fat) |
| Cultured milk /yogurt | Fermented dairy | Not further defined |
| Low-fat cultured milk/ yogurt | Low-fat fermented dairy | Cultured milk/yogurt (≤1.5% fat) |
| Full-fat cultured milk/yogurt | High-fat fermented dairy | Cultured milk/yogurt (≥3.0% fat) |
| Cheese | cheese | Full-fat (>17% fat), low-fat (≤17% fat), and cottage cheese/quark |
| Low-fat cheese | Low-fat cheese | Low-fat varieties (10–17%) and excluded very-low-fat cheese (i.e., cottage cheese/quark (4% fat)) |
| High-fat cheese | Full-fat cheese | Cheese (>17% fat) |
| *Soedamah-Muthu et al. 2013* [20] |  |  |
| Total dairy | Total dairy | All dairy products, except butter and ice cream |
| High-fat dairy | High-fat dairy | Full-fat cheese, yogurt, milk puddings, whole and Channel Islands milk |
| Low-fat dairy | Low-fat dairy | Cottage cheese, semi-skimmed, skimmed milk and milk-based hot drinks |
| Total milk | Total milk | Whole and low-fat milk |
| Fermented dairy | Fermented dairy | Yogurt and total cheese |
| Cheese | Cheese | Full-fat cheese and cottage |
| Yogurt | Yogurt | Not further defined |
| *Louie et al. 2013* [21] |  |  |
| Total dairy | Total dairy | Whole fat milk, reduced fat/skim milk, low fat cheese, whole fat cheese, reduced fat dairy dessert (e.g., low fat yogurt), and medium fat dairy dessert (e.g., custard and whole fat yogurt). |
| Low/reduced fat dairy | Low-fat dairy | Reduce fat/ skim milk, reduced fat dairy dessert and low fat cheese |
| Whole fat dairy | Full-fat dairy | Whole fat milk, whole fat cheese and medium fat dairy dessert |
| *von Ruesten et al, 2013* [22] |  |  |
| Low-fat dairy | Low-fat dairy | Fat-reduced variants of: milk/milkshake (1.5% fat or less), yogurt, fruit yogurt (1.5% fat or less), soured milk/kefir, curd/curd with herbs |
| High-fat dairy | High-fat dairy | Normal- or high-fat variants of: milk/milkshake, yogurt, fruit yogurt, soured milk/kefir, curd/curd with herbs |
| Low-fat cheese | Low-fat cheese | Fat-reduced variants of: Cream cheese, hard cheese (for example, gouda, Emmental cheese, Tilsiter cheese), soft cheese (for example, camembert, brie, gorgonzola) |
| High-fat cheese | High-fat cheese | Normal- or high-fat variants of: Cream cheese, processed cheese, hard cheese (for example, gouda, Emmental cheese, Tilsiter cheese), soft cheese (for example, camembert, brie, gorgonzola), whipped cream |
| *Van Aerde et al, 2013* [23] |  |  |
| Total dairy | Total dairy | Includes all dairy products, except butter |
| High-fat dairy | High-fat dairy | All milk products with a fat content>2.0/100 g or cheese products with a fat content>20 g/100 g |
| Low-fat dairy | Low-fat dairy | All milk products with a fat content<2.0/100 g or cheese products with a fat content<20/100 g |
| Milk | Milk | All milk: skimmed, semi-skimmed, and whole milk |
| Fermented dairy | Fermented dairy | All fermented products, such as yogurt, buttermilk, curds, and cheese products |
| Cheese | Cheese | Soft cheese and hard cheese (both low-fat and high-fat) |
| *Michaelsson et al. 2014* [24] |  |  |
| Milk | Milk | Not further defined |
| Cheese | Cheese | Not further defined |
| Fermented milk products | Fermented dairy | Yogurt and other soured milk products |
| *Praagman et al. 2014* [25] *and Engberink et al. 2009* [13] | |  |
| Total dairy | Total dairy | Milk, buttermilk, yogurt, coffee creamer, curd, pudding, porridge, custard, whipped cream, ice cream, and cheese, but not butter |
| Low-fat dairy | Low-fat dairy | Milk and milk products with a fat content <2.0/100 g and cheese products with a fat content <20/100 g |
| High-fat dairy | High-fat dairy | Milk and milk products with a fat content ≥2.0/100 g and cheese products with a fat content >20/100 g |
| Fermented dairy | Fermented dairy | All types of buttermilk, yogurt, curd and cheese |
| Cheese | Cheese | All types of cheese, excluding curd |
| Yogurt | Yogurt | Not further defined |
| *Haring et al, 2014* [26] |  |  |
| Total dairy intake | Total dairy intake | Not further defined |
| High-fat dairy | High-fat dairy | Not further defined |
| Low-fat dairy | Low-fat dairy | Not further defined |
| *Huang et al. 2015* [27] |  |  |
| Total dairy | Total dairy | Liquid milk and fat-free, low-fat, high-fat, and flavoured dairy products |
| *Bergholdt et al, 2015* [28] |  |  |
| Milk | Milk | whole milk (3.5% fat), semi-skimmed (0.5-1.5% fat) and skimmed milk (0.1-0.3% fat) |
| *Praagman et al. 2015* [29] |  |  |
| Fermented dairy foods | Fermented dairy | Butter milk, yogurt (fat and skim), yogurt drink, curd |
| Yogurt | Yogurt | Not further defined |
| Cheese | Cheese | Cheese 'Goudse', cheese 'Edammer' 40+, cheese 'Leidse', cheese 'brie' 50+, cheese 'Trenta', cheese on pizza |
| *Wang et al. 2015* [30] |  |  |
| Milk | Milk | Not further defined |

Supplemental Table 3. Association between dairy foods and all-cause mortality by subgroups^1^.

| Dairy food | Subgroup | No. study populations | Relative risk | Heterogeneity test | |
| --- | --- | --- | --- | --- | --- |
|  |  |  | (95% CI)^2^ | I² (%) | P-value |
| Total dairy | Overall | 10 | 0.99 (0.96, 1.03) | 62.2 | 0.005 |
| Per 200 g/d | Age (y) |  |  |  |  |
|  | ≤50 | 3 | 0.90 (0.73, 1.11) | 62.4 | 0.070 |
|  | >50 | 7 | 0.99 (0.96, 1.04) | 67.4 | 0.005 |
|  | Follow-up time (y) |  |  |  |  |
|  | ≤10 | 5 | 1.02 (0.99, 1.05) | 51.6 | 0.083 |
|  | >10 | 5 | 0.93 (0.87, 1.01) | 54.0 | 0.069 |
|  | Gender |  |  |  |  |
|  | Men | 1 | 1.01 (0.99, 1.03) |  |  |
|  | Women | 1 | 1.02 (0.99, 1.06) |  |  |
|  | Men and Women | 8 | 0.96 (0.90, 1.03) | 65.5 | 0.005 |
|  | Continent |  |  |  |  |
|  | Europe | 8 | 1.00 (0.97, 1.03) | 62.0 | 0.010 |
|  | Australia | 1 | 0.80 (0.65, 1.00) |  | |
|  | Asia | 1 | 0.69 (0.35, 1.33) |  | |
|  | Confounding factors^2^ | | | | |
|  | Yes | 6 | 0.99 (0.96, 1.03) | 61.3 | 0.024 |
|  | No | 4 | 0.95 (0.76, 1.21) | 70.1 | 0.018 |
|  | BMI |  |  |  |  |
|  | ≤25 | 3 | 1.01 (1.00, 1.03) | 0.0 | 0.458 |
|  | >25 | 6 | 0.95 (0.89, 1.01) | 65.5 | 0.013 |
|  | Newcastle-Ottawa quality score | |  |  |  |
|  | <7 | 3 | 0.78 (0.42, 1.45) | 76.9 | 0.013 |
|  | ≥7 | 7 | 0.99 (0.96, 1.02) | 57.9 | 0.027 |
| Low-fat dairy | Overall | 7 | 1.01 (0.99, 1.03) | 0.0 | 0.734 |
| Per 200 g/d | Age (y) |  |  |  |  |
|  | ≤50 | 2 | 1.01 (0.95, 1.08) | 0.0 | 0.753 |
|  | >50 | 5 | 1.01 (0.99, 1.03) | 0.0 | 0.483 |
|  | Follow-up time (y) |  |  |  |  |
|  | ≤10 | 3 | 1.02 (0.99, 1.04) | 0.0 | 0.813 |
|  | >10 | 4 | 0.97 (0.93, 1.02) | 0.0 | 0.896 |
|  | Gender |  |  |  |  |
|  | Men | 1 | 1.01 (0.98, 1.04) |  |  |
|  | Women | 1 | 1.03 (0.99, 1.06) |  |  |
|  | Men and Women | 5 | 0.99 (0.95, 1.02) | 0.0 | 0.823 |
|  | Continent |  |  |  |  |
|  | Europe | 6 | 1.01 (0.99, 1.03) | 0.0 | 0.624 |
|  | Australia | 1 | 0.97 (0.74, 1.27) |  |  |
|  | Confounding factors^2^ | | | | |
|  | Yes | 6 | 1.01 (0.99,1.03) | 0.0 | 0.816 |
|  | No | 1 | 0.98 (0.92, 1.03) |  |  |
|  | BMI |  |  |  |  |
|  | ≤25 | 2 | 1.02 (0.99, 1.04) | 0.0 | 0.522 |
|  | >25 | 5 | 0.99 (0.95, 1.02) | 0.0 | 0.823 |
|  | Newcastle-Ottawa quality score | |  |  |  |
|  | <7 | 0 |  |  |  |
|  | ≥7 | 7 | 1.01 (0.99, 1.03) | 0.0 | 0.734 |
| Milk | Overall | 12 | 1.00 (0.93, 1.07) | 97.4 | <0.001 |
| Per 244 g/d | Age (y) |  |  |  |  |
|  | ≤50 | 3 | 0.95 (0.92, 0.98) | 0.0 | 0.479 |
|  | >50 | 8 | 1.02 (0.93, 1.11) | 97.8 | <0.001 |
|  | Follow-up time (y) |  |  |  |  |
|  | ≤10 | 0 |  | | |
|  | >10 | 12 | 1.00 (0.93, 1.07) | 97.4 | <0.001 |
|  | Gender |  |  |  |  |
|  | Men | 4 | 0.99 (0.94, 1.04) | 85.9 | <0.001 |
|  | Women | 2 | 1.09 (0.83, 1.43) | 98.6 | <0.001 |
|  | Men and Women | 6 | 0.99 (0.96, 1.03) | 43.4 | 0.116 |
|  | Continent |  |  |  |  |
|  | Europe | 7 | 1.01 (0.91, 1.13) | 98.2 | <0.001 |
|  | Australia | 1 | 0.91 (0.68, 1.22) |  | |
|  | Asia | 2 | 0.95 (0.91, 0.99) | 0.0 | 0.934 |
|  | USA | 2 | 1.01 (0.98, 1.05) | 70.9 | 0.064 |
|  | Confounding factors^2^ | | | | |
|  | Yes | 5 | 1.03 (0.93, 1.07) | 98.3 | <0.001 |
|  | No | 7 | 0.98 (0.95, 1.01) | 71.2 | 0.002 |
|  | BMI |  |  |  |  |
|  | ≤25 | 5 | 1.01 (0.88, 1.16) | 98.3 | <0.001 |
|  | >25 | 6 | 0.99 (0.94, 1.04) | 71.8 | 0.003 |
|  | Newcastle-Ottawa quality score | |  |  |  |
|  | <7 | 6 | 0.97 (0.94, 1.01) | 74.7 | 0.001 |
|  | ≥7 | 6 | 1.04 (0.92,1.17) | 97.9 | <0.001 |
| Total fermented dairy | Overall | 19 | 0.98 (0.97, 0.99) | 94.4 | <0.001 |
| Per 20 g/d | Age (y) |  |  |  |  |
|  | ≤50 | 6 | 1.00 (0.99, 1.00) | 0.0 | 0.816 |
|  | >50 | 12 | 0.97 (0.96, 0.99) | 96.5 | <0.001 |
|  | Follow-up time (y) |  |  |  |  |
|  | ≤10 | 6 | 0.99 (0.99, 1.00) | 59.7 | 0.030 |
|  | >10 | 13 | 0.97 (0.96, 0.99) | 96.1 | <0.001 |
|  | Gender |  |  |  |  |
|  | Men | 4 | 0.99 (0.98, 1.00) | 72.0 | 0.013 |
|  | Women | 4 | 0.96 (0.92, 1.00) | 99.0 | <0.001 |
|  | Men and Women | 11 | 0.99 (0.99, 1.00) | 20.3 | 0.250 |
|  | Continent |  |  |  |  |
|  | Europe | 16 | 0.98 (0.97, 0.99) | 95.3 | <0.001 |
|  | Australia | 2 | 1.06 (0.95, 1.18) | 0.0 | 0.389 |
|  | USA | 1 | 0.99 (0.94, 1.04) |  | |
|  | Confounding factors^2^ | | | | |
|  | Yes | 15 | 0.98 (0.97, 0.99) | 95.6 | <0.001 |
|  | No | 4 | 0.97 (0.94, 1.01) | 0.0 | 0.609 |
|  | BMI |  |  |  |  |
|  | ≤25 | 7 | 0.97 (0.95, 1.00) | 98.0 | <0.001 |
|  | >25 | 11 | 0.99 (0.99, 1.00) | 42.2 | 0.068 |
|  | Newcastle-Ottawa quality score | |  |  |  |
|  | <7 | 3 | 0.99 (0.95, 1.04) | 0.0 | 0.805 |
|  | ≥7 | 16 | 0.98 (0.97, 0.99) | 95.3 | <0.001 |
| Cheese | Overall | 13 | 0.99 (0.96, 1.01) | 93.3 | <0.001 |
| Per 10 g/d | Age (y) |  |  |  |  |
|  | ≤50 | 4 | 1.00 (0.98, 1.02) | 0.0 | 0.784 |
|  | >50 | 8 | 0.98 (0.95, 1.01) | 95.8 | <0.001 |
|  | Follow-up time (y) |  |  |  |  |
|  | ≤10 | 4 | 1.00 (0.99, 1.02) | 21.1 | 0.284 |
|  | >10 | 9 | 0.98 (0.96, 1.01) | 93.4 | <0.001 |
|  | Gender |  |  |  |  |
|  | Men | 2 | 1.00 (0.98, 1.02) | 88.5 | 0.003 |
|  | Women | 2 | 0.96 (0.91, 1.02) | 97.5 | <0.001 |
|  | Men and Women | 9 | 0.99 (0.98, 1.00) | 0.0 | 0.918 |
|  | Continent |  |  |  |  |
|  | Europe | 11 | 0.99 (0.96, 1.01) | 94.3 | <0.001 |
|  | Australia | 1 | 0.96 (0.83, 1.13) |  |  |
|  | USA | 1 | 0.99 (0.97, 1.02) |  |  |
|  | Confounding factors^2^ | | | | |
|  | Yes | 9 | 0.99 (0.96, 1.01) | 95.4 | <0.001 |
|  | No | 4 | 0.99 (0.97, 1.00) | 0.0 | 0.600 |
|  | BMI |  |  |  |  |
|  | ≤25 | 4 | 0.99 (0.94, 1.03) | 97.8 | <0.001 |
|  | >25 | 8 | 0.99 (0.98, 1.00) | 0.0 | 0.906 |
|  | Newcastle-Ottawa quality score | |  |  |  |
|  | <7 | 2 | 0.99 (0.97, 1.02) | 0.0 | 0.675 |
|  | ≥7 | 11 | 0.99 (0.96, 1.01) | 94.3 | <0.001 |

^1^ Insufficient studies to split results for high-fat dairy and yogurt.

^2^ Confounding factors adjusted for age, sex, BMI, smoking, alcohol, leisure activity and total energy intake.

Supplemental Table 4. Association between dairy foods and CHD by subgroups^1^.

| Dairy food | Subgroup | No. study populations | Relative risk | Heterogeneity test | |
| --- | --- | --- | --- | --- | --- |
|  |  |  | (95% CI)^2^ | I² (%) | P-value |
| Total dairy | Overall | 12 | 0.99 (0.96, 1.02) | 38.9 | 0.081 |
| Per 200 g/d | Age (y) |  |  |  |  |
|  | ≤50 | 4 | 1.02 (0.98, 1.06) | 33.1 | 0.214 |
|  | >50 | 8 | 0.97 (0.94, 1.00) | 16.5 | 0.300 |
|  | Follow-up time (y) |  |  |  |  |
|  | ≤10 | 5 | 0.99 (0.96, 1.02) | 0.0 | 0.733 |
|  | >10 | 7 | 0.99 (0.94, 1.03) | 62.4 | 0.014 |
|  | Gender |  |  |  |  |
|  | Men | 2 | 0.99 (0.95, 1.03) | 0.0 | 0.403 |
|  | Women | 4 | 1.00 (0.94, 1.06) | 70.2 | 0.018 |
|  | Men and Women | 6 | 0.98 (0.94, 1.02) | 21.6 | 0.272 |
|  | Continent |  |  |  |  |
|  | Europe | 7 | 0.98 (0.95, 1.02) | 14.9 | 0.317 |
|  | Australia | 1 | 0.87 (0.77, 0.98) |  | |
|  | USA | 4 | 1.01 (0.97, 1.04) | 30.8 | 0.227 |
|  | Confounding factors^2^ | | | | |
|  | Yes | 9 | 1.01 (0.98, 1.03) | 3.4 | 0.406 |
|  | No | 3 | 0.94 (0.88, 1.00) | 37.7 | 0.201 |
|  | BMI |  |  |  |  |
|  | ≤25 | 2 | 0.99 (0.89, 1.10) | 88.8 | 0.003 |
|  | >25 | 10 | 0.99 (0.96, 1.01) | 0.0 | 0.487 |
|  | Newcastle-Ottawa quality score | |  |  |  |
|  | <7 | 1 | 0.94 (0.89, 0.99) |  |  |
|  | ≥7 | 11 | 1.00 (0.97, 1.03) | 25.8 | 0.198 |
| High-fat dairy | Overall | 9 | 0.99 (0.93, 1.05) | 22.9 | 0.240 |
| Per 200 g/d | Age (y) |  |  |  |  |
|  | ≤50 | 2 | 0.89 (0.61, 1.29) | 76.1 | 0.041 |
|  | >50 | 7 | 0.98 (0.92, 1.05) | 0.0 | 0.474 |
|  | Follow-up time (y) |  |  |  |  |
|  | ≤10 | 3 | 0.94 (0.77, 1.14) | 39.1 | 0.194 |
|  | >10 | 6 | 1.00 (0.93, 1.07) | 25.0 | 0.246 |
|  | Gender |  |  |  |  |
|  | Men | 1 | 0.98 (0.88, 1.09) |  |  |
|  | Women | 2 | 1.03 (0.97, 1.10) | 0.0 | 0.800 |
|  | Men and Women | 6 | 0.92 (0.80, 1.06) | 40.3 | 0.137 |
|  | Continent |  |  |  |  |
|  | Europe | 4 | 0.86 (0.68, 1.08) | 30.9 | 0.227 |
|  | Australia | 1 | 0.86 (0.70, 1.05) |  |  |
|  | USA | 4 | 1.02 (0.97, 1.08) | 0.0 | 0.793 |
|  | Confounding factors^2^ | | | | |
|  | Yes | 7 | 1.00 (0.94, 1.07) | 25.5 | 0.235 |
|  | No | 2 | 0.89 (0.75, 1.07) | 0.0 | 0.451 |
|  | BMI |  |  |  |  |
|  | ≤25 | 1 | 1.04 (0.97, 1.11) |  |  |
|  | >25 | 8 | 0.97 (0.89, 1.05) | 19.7 | 0.274 |
|  | Newcastle-Ottawa quality score | |  |  |  |
|  | ≥7 | 9 | 0.99 (0.93, 1.05) | 22.9 | 0.240 |
| Low-fat dairy | Overall | 10 | 1.00 (0.97, 1.03) | 27.3 | 0.193 |
| Per 200 g/d | Age (y) |  |  |  |  |
|  | ≤50 | 2 | 0.97 (0.91, 1.04) | 38.3 | 0.203 |
|  | >50 | 8 | 1.01 (0.97, 1.05) | 26.8 | 0.215 |
|  | Follow-up time (y) |  |  |  |  |
|  | ≤10 | 4 | 1.00 (0.96, 1.06) | 14.5 | 0.320 |
|  | >10 | 6 | 1.00 (0.95, 1.05) | 43.5 | 0.115 |
|  | Gender |  |  |  |  |
|  | Men | 2 | 1.01 (0.97, 1.05) | 0.0 | 0.848 |
|  | Women | 2 | 1.00 (0.87, 1.15) | 75.1 | 0.045 |
|  | Men and Women | 6 | 1.00 (0.95, 1.05) | 35.7 | 0.169 |
|  | Continent |  |  |  |  |
|  | Europe | 6 | 1.02 (0.97, 1.08) | 32.7 | 0.191 |
|  | Australia | 1 | 0.94 (0.73, 1.21) |  |  |
|  | USA | 3 | 0.98 (0.93, 1.03) | 39.6 | 0.191 |
|  | Confounding factors^2^ | | | | |
|  | Yes | 8 | 0.99 (0.96, 1.03) | 34.4 | 0.154 |
|  | No | 2 | 1.04 (0.96, 1.12) | 0.0 | 0.407 |
|  | BMI |  |  |  |  |
|  | ≤25 | 3 | 1.00 (0.92, 1.09) | 54.0 | 0.114 |
|  | >25 | 7 | 1.00 (0.97, 1.04) | 24.9 | 0.239 |
|  | Newcastle-Ottawa quality score | |  |  |  |
|  | ≥7 | 10 | 1.00 (0.97, 1.03) | 27.3 | 0.193 |
| Milk | Overall | 12 | 1.01 (0.96, 1.06) | 45.5 | 0.043 |
| Per 244 g/d | Age (y) |  |  |  |  |
|  | ≤50 | 4 | 1.02 (0.94, 1.12) | 54.6 | 0.086 |
|  | >50 | 8 | 1.00 (0.94, 1.07) | 48.5 | 0.059 |
|  | Follow-up time (y) |  |  |  |  |
|  | ≤10 | 2 | 0.97 (0.91, 1.04) | 0.0 | 0.676 |
|  | >10 | 10 | 1.02 (0.60, 1.08) | 53.1 | 0.024 |
|  | Gender |  |  |  |  |
|  | Men | 4 | 0.98 (0.93, 1.04) | 34.4 | 0.206 |
|  | Women | 3 | 1.04 (0.93, 1.17) | 64.2 | 0.061 |
|  | Men and Women | 5 | 1.00 (0.93, 1.08) | 9.0 | 0.355 |
|  | Continent |  |  |  |  |
|  | Europe | 8 | 1.00 (0.95, 1.04) | 25.4 | 0.227 |
|  | Asia | 2 | 0.99 (0.19, 5.05) | 87.2 | 0.005 |
|  | USA | 2 | 1.03 (0.97, 1.08) | 23.1 | 0.254 |
|  | Confounding factors^2^ | | | | |
|  | Yes | 4 | 1.02 (0.97, 1.08) | 20.3 | 0.288 |
|  | No | 8 | 1.00 (0.93, 1.08) | 54.3 | 0.032 |
|  | BMI |  |  |  |  |
|  | ≤25 | 6 | 1.08 (0.97, 1.20) | 46.0 | 0.099 |
|  | >25 | 5 | 0.98 (0.94, 1.01) | 0.0 | 0.537 |
|  | Newcastle-Ottawa quality score | |  |  |  |
|  | <7 | 5 | 1.01 (0.95, 1.07) | 43.3 | 0.133 |
|  | ≥7 | 7 | 1.00 (0.93, 1.09) | 52.6 | 0.049 |
| Total fermented dairy | Overall | 14 | 0.99 (0.98, 1.01) | 44.6 | 0.037 |
| Per 20 g/d | Age (y) |  |  |  |  |
|  | ≤50 | 5 | 1.00 (0.97, 1.04) | 65.7 | 0.020 |
|  | >50 | 9 | 0.99 (0.98, 1.00) | 21.9 | 0.249 |
|  | Follow-up time (y) |  |  |  |  |
|  | ≤10 | 6 | 1.00 (0.98, 1.02) | 9.6 | 0.355 |
|  | >10 | 8 | 0.99 (0.98, 1.01) | 53.3 | 0.036 |
|  | Gender |  |  |  |  |
|  | Men | 2 | 0.92 (0.77, 1.10) | 57.3 | 0.126 |
|  | Women | 4 | 0.99 (0.97, 1.01) | 53.7 | 0.090 |
|  | Men and Women | 8 | 1.00 (0.98, 1.02) | 41.3 | 0.103 |
|  | Continent |  |  |  |  |
|  | Europe | 14 | 0.99 (0.98, 1.01) | 44.6 | 0.037 |
|  | Confounding factors^2^ | | | | |
|  | Yes | 9 | 1.00 (0.99, 1.01) | 0.0 | 0.447 |
|  | No | 5 | 0.99 (0.97, 1.02) | 72.5 | 0.006 |
|  | BMI |  |  |  |  |
|  | ≤25 | 8 | 0.99 (0.96, 1.02) | 63.2 | 0.008 |
|  | >25 | 6 | 1.00 (0.99, 1.01) | 0.0 | 0.727 |
|  | Newcastle-Ottawa quality score | |  |  |  |
|  | <7 | 4 | 0.99 (0.96, 1.03) | 78.5 | 0.003 |
|  | ≥7 | 10 | 1.00 (0.99, 1.01) | 0.0 | 0.535 |
| Cheese | Overall | 10 | 0.99 (0.97, 1.02) | 40.3 | 0.089 |
| Per 10 g/d | Age (y) |  |  |  |  |
|  | ≤50 | 4 | 1.06 (0.98, 1.15) | 61.7 | 0.05 |
|  | >50 | 6 | 0.99 (0.98, 0.99) | 0.0 | 0.809 |
|  | Follow-up time (y) |  |  |  |  |
|  | ≤10 | 4 | 0.99 (0.95, 1.02) | 27.1 | 0.249 |
|  | >10 | 6 | 1.01 (0.97, 1.05) | 54.4 | 0.052 |
|  | Gender |  |  |  |  |
|  | Men | 1 | 0.97 (0.93, 1.02) |  |  |
|  | Women | 2 | 0.99 (0.98. 1.00) | 0.0 | 0.648 |
|  | Men and Women | 7 | 1.01 (0.97, 1.06) | 48.9 | 0.068 |
|  | Continent |  |  |  |  |
|  | Europe | 10 | 0.99 (0.97, 1.02) | 40.3 | 0.089 |
|  | Confounding factors^2^ | | | | |
|  | Yes | 6 | 0.99 (0.96, 1.01) | 0.0 | 0.461 |
|  | No | 4 | 1.03 (0.97, 1.10) | 71.2 | 0.015 |
|  | BMI |  |  |  |  |
|  | ≤25 | 5 | 1.00 (0.95, 1.05) | 63.3 | 0.028 |
|  | >25 | 5 | 1.00 (0.97, 1.03) | 0.0 | 0.543 |
|  | Newcastle-Ottawa quality score | |  |  |  |
|  | <7 | 3 | 1.14 (0.93, 1.40) | 80.3 | 0.006 |
|  | ≥7 | 7 | 0.99 (0.97, 1.01) | 0.0 | 0.561 |

^1^ Insufficient studies to split results for high-fat dairy and yogurt.

^2^ Confounding factors adjusted for age, sex, BMI, smoking, alcohol, leisure activity and total energy intake.

Supplemental Table 5. Association between dairy foods and CVD by subgroups^1^.

| Dairy food | Subgroup | No. study populations | Relative risk | Heterogeneity test | |
| --- | --- | --- | --- | --- | --- |
|  |  |  | (95% CI)^2^ | I² (%) | P-value |
| Total dairy | Overall | 8 | 0.97 (0.91, 1.02) | 59.9 | 0.015 |
| Per 200 g/d | Age (y) |  |  |  |  |
|  | ≤50 | 3 | 0.68 (0.39, 1.19) | 77.5 | 0.012 |
|  | >50 | 5 | 0.97 (0.91, 1.04) | 52.6 | 0.077 |
|  | Follow-up time (y) |  |  |  |  |
|  | ≤10 | 2 | 1.08 (0.81, 1.44) | 59.4 | 0.116 |
|  | >10 | 6 | 0.95 (0.87, 1.03) | 64.9 | 0.014 |
|  | Gender |  |  |  |  |
|  | Men and Women | 8 | 0.97 (0.91, 1.02) | 59.9 | 0.015 |
|  | Continent |  |  |  |  |
|  | Europe | 5 | 0.98 (0.94, 1.02) | 36.5 | 0.178 |
|  | Australia | 2 | 0.81 (0.60, 1.09) | 52.1 | 0.149 |
|  | Asia | 1 | 0.19 (0.04, 0.76) |  | |
|  | Confounding factors^2^ | | | | |
|  | Yes | 4 | 0.98 (0.92, 1.03) | 58.3 | 0.066 |
|  | No | 4 | 0.95 (0.80, 1.12) | 68.1 | 0.024 |
|  | BMI |  |  |  |  |
|  | ≤25 | 1 | 0.19 (0.04, 0.76) |  |  |
|  | >25 | 7 | 0.97 (0.92, 1.01) | 50.9 | 0.057 |
|  | Newcastle-Ottawa quality score | |  |  |  |
|  | <7 | 2 | 0.56 (0.91, 1.02) | 59.9 | 0.015 |
|  | ≥7 | 6 | 0.96 (0.92, 1.01) | 47.7 | 0.088 |
| High-fat dairy | Overall | 7 | 0.93 (0.84, 1.03) | 37.4 | 0.143 |
| Per 200 g/d | Age (y) |  |  |  |  |
|  | ≤50 | 3 | 0.76 (0.59, 0.97) | 31.5 | 0.232 |
|  | >50 | 4 | 0.99 (0.93, 1.04) | 0.0 | 0.797 |
|  | Follow-up time (y) |  |  |  |  |
|  | ≤10 | 2 | 0.82 (0.67, 1.01) | 0.0 | 0.570 |
|  | >10 | 5 | 0.96 (0.86, 1.07) | 39.4 | 0.159 |
|  | Gender |  |  |  |  |
|  | Men and Women | 7 | 0.93 (0.84, 1.03) | 37.4 | 0.143 |
|  | Continent |  |  |  |  |
|  | Europe | 5 | 0.98 (0.93, 1.04) | 0.0 | 0.450 |
|  | Australia | 2 | 0.72 (0.41, 1.28) | 75.1 | 0.045 |
|  | Confounding factors^2^ | | | | |
|  | Yes | 5 | 0.88 (0.74, 1.04) | 53.9 | 0.070 |
|  | No | 2 | 0.97 (0.85, 1.11) | 0.0 | 0.338 |
|  | BMI |  |  |  |  |
|  | ≤25 | 1 | 0.86 (0.66, 1.13) |  | |
|  | >25 | 6 | 0.94 (0.83, 1.05) | 43.6 | 0.115 |
|  | Newcastle-Ottawa quality score | |  |  |  |
|  | ≥7 | 7 | 0.93 (0.84, 1.03) | 37.4 | 0.143 |
| Low-fat dairy | Overall | 7 | 0.98 (0.95, 1.01) | 0.0 | 0.769 |
| Per 200 g/d | Age (y) |  |  |  |  |
|  | ≤50 | 3 | 0.99 (0.94, 1.04) | 0.0 | 0.577 |
|  | >50 | 4 | 0.97 (0.93, 1.01) | 0.0 | 0.584 |
|  | Follow-up time (y) |  |  |  |  |
|  | ≤10 | 2 | 0.98 (0.94, 1.03) | 0.0 | 0.599 |
|  | >10 | 5 | 0.97 (0.94, 1.01) | 0.0 | 0.579 |
|  | Gender |  |  |  |  |
|  | Men and Women | 7 | 0.98 (0.95, 1.01) | 0.0 | 0.769 |
|  | Continent |  |  |  |  |
|  | Europe | 5 | 0.98 (0.95, 1.01) | 0.0 | 0.888 |
|  | Australia | 2 | 0.96 (0.69, 1.35) | 43.8 | 0.182 |
|  | Confounding factors^2^ | | | | |
|  | Yes | 5 | 0.98 (0.95, 1.01) | 0.0 | 0.742 |
|  | No | 2 | 0.95 (0.86, 1.04) | 0.0 | 0.345 |
|  | BMI |  |  |  |  |
|  | >25 | 6 | 0.97 (0.94, 1.00) | 0.0 | 0.715 |
|  | Newcastle-Ottawa quality score | |  |  |  |
|  | ≥7 | 7 | 0.98 (0.95, 1.01) | 0.0 | 0.769 |
| Milk | Overall | 12 | 1.01 (0.93, 1.10) | 92.4 | <0.001 |
| Per 244 g/d | Age (y) |  |  |  |  |
|  | ≤50 | 2 | 0.96 (0.92, 1.01) | 0.0 | 0.399 |
|  | >50 | 10 | 1.03 (0.93, 1.13) | 92.5 | <0.001 |
|  | Follow-up time (y) |  |  |  |  |
|  | ≤10 | 1 | 1.67 (0.75, 3.72) |  |  |
|  | >10 | 11 | 1.01 (0.92, 1.09) | 93.0 | <0.001 |
|  | Gender |  |  |  |  |
|  | Men | 5 | 0.97 (0.91, 1.05) | 80.2 | <0.001 |
|  | Women | 2 | 0.99 (0.76, 1.28) | 93.4 | <0.001 |
|  | Men and Women | 4 | 1.11 (0.84, 1.47) | 91.2 | 0.052 |
|  | Continent |  |  |  |  |
|  | Europe | 7 | 1.06 (0.96, 1.17) | 95.0 | <0.001 |
|  | Australia | 1 | 0.77 (0.46, 1.29) |  |  |
|  | Asia | 4 | 0.91 (0.82, 1.02) | 39.8 | 0.173 |
|  | Confounding factors^2^ | | | | |
|  | Yes | 5 | 1.10 (0.98-1.25) | 95.3 | <0.001 |
|  | No | 7 | 0.94 (0.89-0.99) | 28.6 | 0.210 |
|  | BMI |  |  |  |  |
|  | ≤25 | 5 | 0.99 (0.80, 1.22) | 93.5 | <0.001 |
|  | >25 | 7 | 1.00 (0.94, 1.06) | 70.9 | 0.002 |
|  | Newcastle-Ottawa quality score | |  |  |  |
|  | <7 | 4 | 0.95 (0.90, 1.00) | 22.1 | 0.278 |
|  | ≥7 | 8 | 1.05 (0.94, 1.16) | 93.0 | <0.001 |
| Total fermented dairy | Overall | 17 | 0.98 (0.97, 0.99) | 87.5 | <0.001 |
| Per 20 g/d | Age (y) |  |  |  |  |
|  | ≤50 | 7 | 1.00 (0.99, 1.01) | 0.0 | 0.541 |
|  | >50 | 10 | 0.98 (0.96, 0.99) | 92.5 | <0.001 |
|  | Follow-up time (y) |  |  |  |  |
|  | ≤10 | 5 | 1.00 (0.99, 1.01) | 0.0 | 0.659 |
|  | >10 | 12 | 0.97 (0.96, 0.99) | 91.1 | <0.001 |
|  | Gender |  |  |  |  |
|  | Men | 2 | 0.99 (0.96, 1.01) | 77.3 | 0.036 |
|  | Women | 2 | 0.93 (0.81, 1.06) | 99.0 | <0.001 |
|  | Men and Women | 13 | 0.99 (0.99, 1.00) | 0.0 | 0.476 |
|  | Continent |  |  |  |  |
|  | Europe | 15 | 0.98 (0.97, 0.99) | 88.9 | <0.001 |
|  | Australia | 2 | 0.89 (0.72, 1.10) | 0.0 | 0.510 |
|  | Confounding factors^2^ | | | | |
|  | Yes | 14 | 0.98 (0.97, 0.99) | 89.5 | <0.001 |
|  | No | 3 | 1.02 (0.98, 1.06) | 0.0 | 0.593 |
|  | BMI |  |  |  |  |
|  | ≤25 | 2 | 0.93 (0.81, 1.06) | 99.0 | <0.001 |
|  | >25 | 13 | 0.99 (0.99, 1.00) | 25.0 | 0.191 |
|  | Newcastle-Ottawa quality score | |  |  |  |
|  | <7 | 2 | 1.02 (0.97, 1.08) | 4.1 | 0.307 |
|  | ≥7 | 15 | 0.98 (0.97, 0.99) | 88.8 | <0.001 |
| Cheese | Overall | 11 | 0.98 (0.95, 1.00) | 82.6 | <0.001 |
| Per 10 g/d | Age (y) |  |  |  |  |
|  | ≤50 | 5 | 0.98 (0.96, 1.01) | 0.0 | 0.528 |
|  | >50 | 6 | 0.98 (0.94, 1.01) | 90.5 | <0.001 |
|  | Follow-up time (y) |  |  |  |  |
|  | ≤10 | 4 | 1.00 (0.96, 1.04) | 0.0 | 0.853 |
|  | >10 | 7 | 0.97 (0.85, 1.00) | 88.7 | <0.001 |
|  | Gender |  |  |  |  |
|  | Men | 1 | 0.99 (0.97, 1.00) |  |  |
|  | Women | 1 | 0.93 (0.92, 0.94) |  |  |
|  | Men and Women | 9 | 0.99 (0.97, 1.00) | 0.0 | 0.764 |
|  | Continent |  |  |  |  |
|  | Europe | 10 | 0.98 (0.95, 1.00) | 84.1 | <0.001 |
|  | Australia | 1 | 0.86 (0.65, 1.15) |  |  |
|  | Confounding factors^2^ | | | | |
|  | Yes | 9 | 0.98 (0.95, 1.00) | 85.2 | <0.001 |
|  | No | 2 | 1.00 (0.96, 1.05) | 0.0 | 0.354 |
|  | BMI |  |  |  |  |
|  | ≤25 | 1 | 0.93 (0.92, 0.94) |  |  |
|  | >25 | 8 | 0.99 (0.98, 0.99) | 0.0 | 0.679 |
|  | Newcastle-Ottawa quality score | |  |  |  |
|  | <7 | 1 | 0.95 (0.83, 1.08) |  |  |
|  | ≥7 | 10 | 0.98 (0.95, 1.01) | 84.3 | <0.001 |

^1^ Insufficient studies to split results for high-fat dairy and yogurt.

^2^ Confounding factors adjusted for age, sex, BMI, smoking, alcohol, leisure activity and total energy intake.

Supplemental Figure 1. Forest plot for the association between total dairy intake and all-cause mortality. Squares represent study-specific RRs. Square areas are proportional to the overall specific-study weight to the overall meta-analysis. Horizontal lines represent 95% CIs. Diamonds represent the pooled relative risk and 95% CIs. Overall no association between total dairy and all-cause mortality (per increment of 200 g/d), including 10 populations (n=175,063 individuals). Heterogeneity (I^2^) of between-study variations is 62.2%.

Supplemental Figure 2. Forest plot for the association between total dairy intake and CHD. Squares represent study-specific RRs. Square areas are proportional to the overall specific-study weight to the overall meta-analysis. Horizontal lines represent 95% CIs. Diamonds represent the pooled relative risk and 95% CIs. Overall no association between total dairy and CHD (per increment of 200 g/d), including 12 populations (n=330,350 individuals). Heterogeneity (I^2^) of between-study variations is 38.9%.

Supplemental Figure 3. Forest plot for the association between total dairy intake and CVD. Squares represent study-specific RRs. Square areas are proportional to the overall specific-study weight to the overall meta-analysis. Horizontal lines represent 95% CIs. Diamonds represent the pooled relative risk and 95% CIs. Overall no association between total dairy and CVD (per increment of 200 g/d), including 8 populations (n=76,207 individuals). Heterogeneity (I^2^) of between-study variations is 59.9%.

Supplemental Figure 4. Forest plot for the association between high-fat dairy intake and all-cause mortality. Squares represent study-specific RRs. Square areas are proportional to the overall specific-study weight to the overall meta-analysis. Horizontal lines represent 95% CIs. Diamonds represent the pooled relative risk and 95% CIs. Overall no association between high-fat dairy and all-cause mortality (per increment of 200 g/d), including 5 populations (n=47,126 individuals). Heterogeneity (I^2^) of between-study variations is 0%.

Supplemental Figure 5. Forest plot for the association between high-fat dairy intake and CHD. Squares represent study-specific RRs. Square areas are proportional to the overall specific-study weight to the overall meta-analysis. Horizontal lines represent 95% CIs. Diamonds represent the pooled relative risk and 95% CIs. Overall no association between high-fat dairy and CHD (per increment of 200 g/d), including 9 populations (n=171,627 individuals). Heterogeneity (I^2^) of between-study variations is 22.9%.

Supplemental Figure 6. Forest plot for the association between high-fat dairy intake and CVD. Squares represent study-specific RRs. Square areas are proportional to the overall specific-study weight to the overall meta-analysis. Horizontal lines represent 95% CIs. Diamonds represent the pooled relative risk and 95% CIs. Overall no association between high-fat dairy and CVD (per increment of 200 g/d), including 7 populations (n=95,242 individuals). Heterogeneity (I^2^) of between-study variations is 37.4%.

Supplemental Figure 7. Forest plot for the association between low-fat dairy intake and all-cause mortality. Squares represent study-specific RRs. Square areas are proportional to the overall specific-study weight to the overall meta-analysis. Horizontal lines represent 95% CIs. Diamonds represent the pooled relative risk and 95% CIs. Overall no association between low-fat dairy and all-cause mortality (per increment of 200 g/d), including 7 populations (n=167,978 individuals). Heterogeneity (I^2^) of between-study variations is 0%.

Supplemental Figure 8. Forest plot for the association between low-fat dairy intake and CHD. Squares represent study-specific RRs. Square areas are proportional to the overall specific-study weight to the overall meta-analysis. Horizontal lines represent 95% CIs. Diamonds represent the pooled relative risk and 95% CIs. Overall no association between low-fat dairy and CHD (per increment of 200 g/d), including 10 populations (n=262,228 individuals). Heterogeneity (I^2^) of between-study variations is 27.3%.

Supplemental Figure 9. Forest plot for the association between low-fat dairy intake and CVD. Squares represent study-specific RRs. Square areas are proportional to the overall specific-study weight to the overall meta-analysis. Horizontal lines represent 95% CIs. Diamonds represent the pooled relative risk and 95% CIs. Overall no association between low-fat dairy and CVD (per increment of 200 g/d), including 7 populations (n=95,242 individuals). Heterogeneity (I^2^) of between-study variations is 0%.

Supplemental Figure 10. Forest plot for the association between milk intake and all-cause mortality. Squares represent study-specific RRs. Square areas are proportional to the overall specific-study weight to the overall meta-analysis. Horizontal lines represent 95% CIs. Diamonds represent the pooled relative risk and 95% CIs. Overall no association between milk and all-cause mortality (per increment of 244 g/d), including 12 populations (n=268,570 individuals). Heterogeneity (I^2^) of between-study variations is 97.4%.

Supplemental Figure 11. Forest plot for the association between milk intake and CHD. Squares represent study-specific RRs. Square areas are proportional to the overall specific-study weight to the overall meta-analysis. Horizontal lines represent 95% CIs. Diamonds represent the pooled relative risk and 95% CIs. Overall no association between milk and CHD (per increment of 244 g/d), including 12 populations (n=230,621 individuals). Heterogeneity (I^2^) of between-study variations is 45.5%.

Supplemental Figure 12. Forest plot for the association between milk intake and CVD. Squares represent study-specific RRs. Square areas are proportional to the overall specific-study weight to the overall meta-analysis. Horizontal lines represent 95% CIs. Diamonds represent the pooled relative risk and 95% CIs. Overall no association between milk and CVD (per increment of 244 g/d), including 12 populations (n=249,779 individuals). Heterogeneity (I^2^) of between-study variations is 92.4%.

Supplemental Figure 13. Forest plot for the association between total fermented dairy intake and CHD. Squares represent study-specific RRs. Square areas are proportional to the overall specific-study weight to the overall meta-analysis. Horizontal lines represent 95% CIs. Diamonds represent the pooled relative risk and 95% CIs. Overall no association between total fermented dairy and CHD (per increment of 20 g/d), including 14 populations (n=256,091 individuals). Heterogeneity (I^2^) of between-study variations is 44.6%.

Supplemental Figure 14. Forest plot for the association between cheese intake and all-cause mortality. Squares represent study-specific RRs. Square areas are proportional to the overall specific-study weight to the overall meta-analysis. Horizontal lines represent 95% CIs. Diamonds represent the pooled relative risk and 95% CIs. Overall no association between cheese and all-cause mortality (per increment of 10 g/d), including 13 populations (n=342,120 individuals). Heterogeneity (I^2^) of between-study variations is 93.3%.

Supplemental Figure 15. Forest plot for the association between cheese intake and CHD. Squares represent study-specific RRs. Square areas are proportional to the overall specific-study weight to the overall meta-analysis. Horizontal lines represent 95% CIs. Diamonds represent the pooled relative risk and 95% CIs. Overall no association between cheese and CHD (per increment of 10 g/d), including 10 populations (n=256,091 individuals). Heterogeneity (I^2^) of between-study variations is 40.3%.

Supplemental Figure 16. Forest plot for the association between yogurt intake and all-cause mortality. Squares represent study-specific RRs. Square areas are proportional to the overall specific-study weight to the overall meta-analysis. Horizontal lines represent 95% CIs. Diamonds represent the pooled relative risk and 95% CIs. Overall no association between yogurt and all-cause mortality (per increment of 50 g/d), including 3 populations (n=40,460 individuals). Heterogeneity (I^2^) of between-study variations is 65.8%.

Supplemental Figure 17. Forest plot for the association between yogurt intake and CHD. Squares represent study-specific RRs. Square areas are proportional to the overall specific-study weight to the overall meta-analysis. Horizontal lines represent 95% CIs. Diamonds represent the pooled relative risk and 95% CIs. Overall no association between yogurt and CHD (per increment of 50 g/d), including 3 populations (n=98,936 individuals). Heterogeneity (I^2^) of between-study variations is 0%.

Supplemental Figure 18. Forest plot for the association between yogurt intake and CVD. Squares represent study-specific RRs. Square areas are proportional to the overall specific-study weight to the overall meta-analysis. Horizontal lines represent 95% CIs. Diamonds represent the pooled relative risk and 95% CIs. Overall no association between yogurt and CVD (per increment of 50 g/d), including 3 populations (n=36,624individuals). Heterogeneity (I^2^) of between-study variations is 0%.

Supplemental Figure 19. Funnel plot for studies of the association between total dairy intake and all-cause mortality based on linear dose-response slopes (no. of cases=21,222; total n=175,063). Each dot indicates a study population with its relative risk (RR). The y-axis represents the SEs of the log (RR). Test for publication bias: Egger’s test *P* = 0.086, symmetry indicates no evidence of publication bias.

Supplemental Figure 20. Funnel plot for studies of the association between total dairy intake and CHD based on linear dose-response slopes (no. of cases=8,298; total n=330,350). Each dot indicates a study population with its relative risk (RR). The y-axis represents the SEs of the log (RR). Test for publication bias: Egger’s test *P* = 1.000, symmetry indicates no evidence of publication bias.

Supplemental Figure 21. Funnel plot for studies of the association between low-fat dairy intake and CHD based on linear dose-response slopes (no. of cases=6,244; total n=262,228). Each dot indicates a study population with its relative risk (RR). The y-axis represents the SEs of the log (RR). Test for publication bias: Egger’s test *P* = 0.747, symmetry indicates no evidence of publication bias.

Supplemental Figure 22. Funnel plot for studies of the association between milk intake and all-cause mortality based on linear dose-response slopes (no. of cases=69,355; total n=268,570). Each dot indicates a study population with its relative risk (RR). The y-axis represents the SEs of the log (RR).Test for publication bias: Egger’s test *P* = 0.254, symmetry indicates no evidence of publication bias.

Supplemental Figure 23. Funnel plot for studies of the association between milk intake and CHD based on linear dose-response slopes (no. of cases=8,612; total n=230,621). Each dot indicates a study population with its relative risk (RR). The y-axis represents the SEs of the log (RR). Test for publication bias: Egger’s test *P* = 0.397, symmetry indicates no evidence of publication bias.

Supplemental Figure 24. Funnel plot for studies of the association between milk intake and CVD based on linear dose-response slopes (no. of cases=21,580; total n=249,779). Each dot indicates a study population with its relative risk (RR). The y-axis represents the SEs of the log (RR). Test for publication bias: Egger’s test *P* = 0.449, symmetry indicates no evidence of publication bias.

Supplemental Figure 25. Funnel plot for studies of the association between fermented dairy intake and CHD based on linear dose-response slopes (no. of cases=5,667; total n=256,091). Each dot indicates a study population with its relative risk (RR). The y-axis represents the SEs of the log (RR). Test for publication bias: Egger’s test *P* = 0.726, symmetry indicates no evidence of publication bias.

Supplemental Figure 26. Funnel plot for studies of the association between cheese intake and all-cause mortality based on linear dose-response slopes (no. of cases=54,125; total n=342,120). Each dot indicates a study population with its relative risk (RR). The y-axis represents the SEs of the log (RR). Test for publication bias: Egger’s test *P* = 0.310, symmetry indicates no evidence of publication bias.

Supplemental Figure 27. Funnel plot for studies of the association between cheese intake and CHD based on linear dose-response slopes (no. of cases=4,022; total n=256,091). Each dot indicates a study population with its relative risk (RR). The y-axis represents the SEs of the log (RR). Test for publication bias: Egger’s test *P* = 0.273, symmetry indicates no evidence of publication bias.

Supplemental Figure 28. Spaghetti plot for the association between total dairy intake and all-cause mortality. Each light blue line represents a study population. Circles are placed at the study-specific RRs that are related to the corresponding quantity of the intake. Circles area is proportional to the study-specific overall weight. Solid red line represents the pooled RR at each quantity of intake and the two dashed dark blue lines are the corresponding 95% CI.

REFERENCES

1. Kahn HA, Phillips RL, Snowdon DA, Choi W. Association between Reported Diet and All-Cause Mortality - 21-Year Follow-up on 27,530 Adult 7th-Day Adventists. Am J Epidemiol. 1984;119:775-87.
2. Mann JI, Appleby PN, Key TJ, Thorogood M. Dietary determinants of ischaemic heart disease in health conscious individuals. Heart.1997;78:450-5.
3. Hu FB, Stampfer MJ, Manson JE, Ascherio A, Colditz GA, Speizer FE, Hennekens CH, Willett WC. Dietary saturated fats and their food sources in relation to the risk of coronary heart disease in women. Am J Clin Nutr. 1999;70:1001-8.
4. Appleby PN, Thorogood M, Mann JI, Key TJ. The Oxford Vegetarian Study: an overview. Am J Clin Nutr. 1999;70:525s-31s.
5. Bostick RM, Kushi LH, Wu Y, Meyer KA, Sellers TA, Folsom AR. Relation of calcium, vitamin D, and dairy food intake to ischemic heart disease mortality among postmenopausal women. Am J Epidemiol. 1999;149:151-61.
6. Fortes C, Forastiere F, Farchi S, Rapiti E, Pastori G, Perucci CA. Diet and overall survival in a cohort of very elderly people. Epidemiology. 2000;11:440-5.
7. Ness AR, Smith GD, Hart C. Milk, coronary heart disease and mortality. J Epidemiol Community Health. 2001;55:379-82.
8. Al-Delaimy WK, Rimm E, Willett WC, Stampfer MJ, Hu FB. A prospective study of calcium intake from diet and supplements and risk of ischemic heart disease among men. Am J Clin Nutr. 2003;77:814-8.
9. Elwood PC, Pickering JE, Fehily AM, Hughes J, Ness AR. Milk drinking, ischaemic heart disease and ischaemic stroke I. Evidence from the Caerphilly cohort. Eur J Clin Nutr. 2004;58:711-7.
10. Knoops KT, Groot de LC, Fidanza F, Alberti-Fidanza A, Kromhout D, van Staveren WA. Comparison of three different dietary scores in relation to 10-year mortality in elderly European subjects: the HALE project. Eur J Clin Nutr. 2006;60:746-55.
11. Paganini-Hill A, Kawas CH, Corrada MM. Non-alcoholic beverage and caffeine consumption and mortality: the Leisure World Cohort Study. Prev Med. 2007;44:305-10.
12. Panagiotakos D, Pitsavos C, Chrysohoou C, Palliou K, Lentzas I, Skoumas I, Stefanadis C. Dietary patterns and 5-year incidence of cardiovascular disease: a multivariate analysis of the ATTICA study. Nutrition, metabolism, and cardiovascular diseases: Nutr Metab Cardiovasc Dis. 2009;19:253-63.
13. Engberink MF, Soedaman-Muthu SS, Boessenkool-Pape J, van Rooij FJA, Hofman A, Witteman JCM, Geleijnse JM. Dairy intake in relation to all-cause mortality and risk of cardiovascular disease: The Rotterdam Study. San Francisco: CA: American Heart Association. 2010:140 (poster 71.).
14. Bonthuis M, Hughes MC, Ibiebele TI, Green AC, van der Pols JC. Dairy consumption and patterns of mortality of Australian adults. Eur J Clin Nutr. 2010;64:569-77.
15. Goldbohm RA, Chorus AM, Galindo Garre F, Schouten LJ, van den Brandt PA. Dairy consumption and 10-y total and cardiovascular mortality: a prospective cohort study in the Netherlands. Am J Clin Nutr. 2011;93:615-27.
16. Sonestedt E, Wirfält E, Wallström P, Gullberg B, Orho-Melander M, Hedblad B. Dairy products and its association with incidence of cardiovascular disease: The Malmö diet and cancer cohort. Eur J Epidemiol. 2011;26:609-18.
17. Kondo I, Ojima T, Nakamura M, Hayasaka S, Hozawa A, Saitoh S, Ohnishi H, Akasaka H, Hayakawa T, Murakami Y, Okuda N, Miura K, Okayama A, Ueshima H. Consumption of dairy products and death from cardiovascular disease in the Japanese general population: the NIPPON DATA80. J Epidemiol. 2013;23:47-54.
18. Dalmeijer GW, Struijk EA, Van Der Schouw YT, Soedamah-Muthu SS, Verschuren WMM, Boer JMA, Geleijnse JM, Beulens JWJ. Dairy intake and coronary heart disease or stroke - A population-based cohort study. Int J Cardiol. 2013;167:925-9.
19. Patterson E, Larsson SC, Wolk A, Akesson A. Association between dairy food consumption and risk of myocardial infarction in women differs by type of dairy food1. J Nutr. 2013;143:74-9.
20. Soedamah-Muthu SS, Masset G, Verberne L, Geleijnse JM, Brunner EJ. Consumption of dairy products and associations with incident diabetes, CHD and mortality in the Whitehall II study. Br J Nutr. 2013;109:718-26.
21. Louie JC, Flood VM, Burlutsky G, Rangan AM, Gill TP, Mitchell P. Dairy consumption and the risk of 15-year cardiovascular disease mortality in a cohort of older Australians. Nutrients. 2013;5:441-54.
22. von Ruesten A, Feller S, Bergmann MM, Boeing H. Diet and risk of chronic diseases: results from the first 8 years of follow-up in the EPIC-Potsdam study. Eur J Clin Nutr. 2013;67:412-9.
23. van Aerde MA, Soedamah-Muthu SS, Geleijnse JM, Snijder MB, Nijpels G, Stehouwer CD, Dekker JM. Dairy intake in relation to cardiovascular disease mortality and all-cause mortality: the Hoorn Study. Eur J Nutr. 2013;52:609-16.
24. Michaelsson K, Wolk A, Langenskiold S, Basu S, Warensjo Lemming E, Melhus H, Byberg L. Milk intake and risk of mortality and fractures in women and men: cohort studies. BMJ. 2014;349:g6015.
25. Praagman J, Franco OH, Ikram MA, Soedamah-Muthu SS, Engberink MF, van Rooij FJ, Hofman A, Geleijnse JM. Dairy products and the risk of stroke and coronary heart disease: the Rotterdam Study. Eur J Nutr. 2015;54:981-90.
26. Haring B, Gronroos N, Nettleton JA, Wyler Von Ballmoos MC, Selvin E, Alonso A. Dietary protein intake and coronary heart disease in a large community based cohort: Results from the Atherosclerosis Risk in Communities (ARIC) study. PLoS ONE. 2014;9:e109552.
27. Huang LY, Wahlqvist ML, Huang YC, Lee MS. Optimal dairy intake is predicated on total, cardiovascular, and stroke mortalities in a Taiwanese cohort. J Am Coll Nutr. 2014;33:426-36.
28. Bergholdt HK, Nordestgaard BG, Varbo A, Ellervi k C. Milk intake is not associated with ischaemic heart disease in observational or Mendelian randomization analyses in 98,529 Danish adults. Int J Epidemiol. 2015;44:587-603.
29. Praagman J, Dalmeijer GW, van der Schouw YT, Soedamah-Muthu SS, Monique Verschuren WM, Bas Bueno-de-Mesquita H, Geleijnse JM, Beulens JW. The relationship between fermented food intake and mortality risk in the European Prospective Investigation into Cancer and Nutrition-Netherlands cohort. Br J Nutr. 2015;113:498-506.
30. Wang C, Yatsuya H, Tamakoshi K, Iso H, Tamakoshi A. Milk drinking and mortality: findings from the Japan collaborative cohort study. J Epidemiol. 2015;25:66-73.
