# Supplementary material for: Milk and dairy consumption and risk of cardiovascular diseases and all-cause mortality: dose–response meta-analysis of prospective cohort studies
Source: Eur J Epidemiol. 2017 Apr 3;32(4):269–87. doi: 10.1007/s10654-017-0243-1 (PMC5437143; doi:10.1007/s10654-017-0243-1)
Supplement: Supplementary file 2 — Supplementary material 2 (DOCX 19 kb) [file 10654_2017_243_MOESM2_ESM.docx]

**Milk and dairy consumption and risk of cardiovascular diseases and all-cause mortality: dose-response meta-analysis of prospective cohort studies.**

Jing Guo^1^, Arne Astrup^2^, Julie A. Lovegrove^3^, Lieke Gijsbers^4^, David I Givens^1^, Sabita S. Soedamah-Muthu^4^

^1^ Centre for Food, Nutrition and Health, University of Reading, RG6 6AR, UK, JG: PhD, jing.guo@pgr.reading.ac.uk. DIG: Professor of Food Chain Nutrition and Head of Centre for Food, Nutrition and Health, d.i.givens@reading.ac.uk.

^2^ Department of Nutrition, Exercise and Sports, University of Copenhagen, DK-2200, Denmark, Professor and Head of Department of Nutrition, Exercise and Sports, ast@nexs.ku.dk.

^3^ Hugh Sinclair Unit of Human Nutrition and Institute for Cardiovascular and Metabolic Research, University of Reading, RG6 6AP, UK, Professor and Hugh Sinclair Chair in Human Nutrition, j.a.lovegrove@reading.ac.uk.

^4^ Division of Human Nutrition, Wageningen University and Research, Wageningen, 6708 WE, the Netherlands. LG: PhD, lieke.gijsbers@wur.nl. SSSM: Assistant Professor, sabita.soedamah-muthu@wur.nl.

Correspondence to: Jing Guo^1^, [jing.guo@pgr.reading.ac.uk](mailto:jing.guo@pgr.reading.ac.uk)

| **Item No** | **Recommendation** | **Reported on Page No** |
| --- | --- | --- |
| Reporting of background should include | | |
| 1 | Problem definition | 4-5 |
| 2 | Hypothesis statement | 4-5 |
| 3 | Description of study outcome(s) | 4-5 |
| 4 | Type of exposure or intervention used | 4-5 |
| 5 | Type of study designs used | 4-5 |
| 6 | Study population | 4-5 |
| Reporting of search strategy should include | | |
| 7 | Qualifications of searchers (eg, librarians and investigators) | 3, 7 |
| 8 | Search strategy, including time period included in the synthesis and key words | 6, Supplemental Methods |
| 9 | Effort to include all available studies, including contact with authors | 6 |
| 10 | Databases and registries searched | 6 |
| 11 | Search software used, name and version, including special features used (eg, explosion) | 6 |
| 12 | Use of hand searching (eg, reference lists of obtained articles) | 6 |
| 13 | List of citations located and those excluded, including justification | 6 |
| 14 | Method of addressing articles published in languages other than English | 6 |
| 15 | Method of handling abstracts and unpublished studies | 6 |
| 16 | Description of any contact with authors | 6 |
| Reporting of methods should include | | |
| 17 | Description of relevance or appropriateness of studies assembled for assessing the hypothesis to be tested | 6 |
| 18 | Rationale for the selection and coding of data (eg, sound clinical principles or convenience) | 6, Figure 1 |
| 19 | Documentation of how data were classified and coded (eg, multiple raters, blinding and interrater reliability) | 6, Supplemental Table 2 |
| 20 | Assessment of confounding (eg, comparability of cases and controls in studies where appropriate) | 7 |
| 21 | Assessment of study quality, including blinding of quality assessors, stratification or regression on possible predictors of study results | 7, Supplemental Methods |
| 22 | Assessment of heterogeneity | 8 |
| 23 | Description of statistical methods (eg, complete description of fixed or random effects models, justification of whether the chosen models account for predictors of study results, dose-response models, or cumulative meta-analysis) in sufficient detail to be replicated | 7-8 |
| 24 | Provision of appropriate tables and graphics | Supplementary files |
| Reporting of results should include | | |
| 25 | Graphic summarizing individual study estimates and overall estimate | Table 1- 2, Figure 1-4, Supplemental online material |
| 26 | Table giving descriptive information for each study included | Table 1 |
| 27 | Results of sensitivity testing (eg, subgroup analysis) | 7-11,  Supplemental Table 3-5 |
| 28 | Indication of statistical uncertainty of findings | 8-12 |

**Meta-analysis of Observational Studies in Epidemiology (MOOSE) Checklist**

| **Item No** | **Recommendation** | **Reported on Page No** |
| --- | --- | --- |
| Reporting of discussion should include | | |
| 29 | Quantitative assessment of bias (eg, publication bias) | 8-9, Supplemental Figure 19-27 |
| 30 | Justification for exclusion (eg, exclusion of non-English language citations) | NA |
| 31 | Assessment of quality of included studies | 16, Supplemental Table 1 |
| Reporting of conclusions should include | | |
| 32 | Consideration of alternative explanations for observed results | 16-17 |
| 33 | Generalization of the conclusions (ie, appropriate for the data presented and within the domain of the literature review) | 16-17 |
| 34 | Guidelines for future research | 16 |
| 35 | Disclosure of funding source | 3 |
